# Biochemical characterization of *Borrelia burgdorferi*’s RecA protein

**DOI:** 10.1371/journal.pone.0187382

**Published:** 2017-10-31

**Authors:** Shu Hui Huang, Madison A. Hart, Matthew Wade, McKayla R. Cozart, Siobhan L. McGrath, Kerri Kobryn

**Affiliations:** Department of Microbiology & Immunology, College of Medicine, University of Saskatchewan, Academic Health Sciences Building, Saskatoon, Saskatchewan, Canada; University of Kentucky College of Medicine, UNITED STATES

## Abstract

RecA plays key roles in DNA recombination, replication and repair. Mutation of *recA* in the Lyme disease spirochete, *Borrelia burgdorferi*, fails to produce some of the phenotypes expected from study of *recA* mutation in other organisms. ‘Missing’ *recA* phenotypes include a lack of growth or viability effects, including in the presence of DNA damage, and a lack of a role in *vlsE* antigenic variation and infectivity. We present a purification and biochemical characterization of recombinant *B*. *burgdorferi* RecA protein. We find that *B*. *burgdorferi* RecA displays the expected properties of being a DNA-dependent ATPase, of having an intrinsic binding preference for ssDNA over dsDNA enhanced by ATP binding, of promoting DNA pairing and strand exchange reactions and of having a detectable coprotease activity with *E*. *coli* LexA repressor. DNA pairing and strand exchange reactions promoted by *B*. *burgdorferi* RecA show an unusually strong dependence upon the presence of the cognate ssDNA binding protein (SSB) but are very sensitive to inhibition by SSB when the ssDNA was prebound by SSB. This indicates *B*. *burgdorferi* RecA may have an enhanced requirement for recombinational mediators to promote RecA-SSB exchange, despite the absence of homologues of the RecF pathway proteins that normally play this role in eubacteria. Finally, we do not find any unusual, intrinsic properties of *B*. *burgdorferi*’s RecA protein to explain the unusual phenotype of *recA* mutation and suggest that there may be alternative recombinase functions that could explain the ‘missing’ phenotypes.

## Introduction

RecA plays key roles in the processes of homologous recombination, DNA damage sensing/repair and the resetting of stalled or collapsed replication forks [1,2,3]. Biochemical characterization of RecA from *E*. *coli* and other eubacteria reveal that RecA is a DNA-dependent ATPase that possesses DNA pairing and strand exchange activities. Additionally, the activated form of RecA (the ATP-bound presynaptic filament formed on ssDNA) senses excessive ssDNA production to signal the presence of DNA damage or invading DNA through activation of a specialized DNA damage response referred to as the SOS response. Activated RecA triggers the SOS response by virtue of its coprotease activity, wherein it chaperones the transcriptional repressor, LexA, to cleave itself, thereby derepressing a suite of ~40 genes involved in the DNA damage response [2,4].

DNA strand exchange promoted by RecA is often described as occurring in three stages: presynapsis, synapsis and branch migration. The presynaptic stage involves ATP-promoted cooperative binding of RecA to ssDNA to produce the presynaptic filament [5,6]. The presynaptic filament promotes all the catalytic steps of strand exchange and possesses coprotease activity. The presynaptic filament binds dsDNA via its secondary binding site and performs the search for homology. Once homology is located, strand exchange commences and heteroduplex DNA is formed in the primary binding site, the outgoing single strand occupies the secondary site [7,8,9,10]. *E*. *coli* RecA and many other RecA-like proteins are able to extend strand exchange over long stretches of DNA in a process called branch migration [11].

Single-stranded DNA binding protein (SSB) also plays a key role in recombination by protecting ssDNA from nucleases, by removing DNA secondary structural barriers to continuous presynaptic filament formation and by stabilizing the ssDNA displaced by strand exchange, preventing RecA binding to this ssDNA followed by reinvasion events that could result in reversal of the strand exchange reaction [12,13,14]. SSB also serves as an assembly platform that recruits DNA replication, recombination and repair proteins to sites of ssDNA [15]. A recent example from the Lyme disease spirochete, *Borrelia burgdorferi*, is demonstration of a physical and functional interaction between the telomere resolvase, ResT, that forms the hairpin telomeres of this organism and purified SSB [16].

RecA function in the spirochete bacterium, *Borrelia burgdorferi*, has been studied by complementation of *E*. *coli recA* mutants and by examination of the phenotypic consequences of *recA* mutation *in vivo* [17,18,19,20,21]. Complementation studies have revealed that *B*. *burgdorferi recA* can fully complement recombination deficiency of the *E*. *coli recA* mutants but are much less effective than *E*. *coli recA* in reversing the sensitivity of *E*. *coli recA* mutants to DNA damaging treatments such as UV light exposure and mitomycin C treatment [17,18]. The Liveris *et al*. study also suggested that *B*. *burgdorferi* RecA possesses an enhanced, DNA damage-independent coprotease activity with λ repressor [18]. Examination of the phenotype of *B*. *burgdorferi recA* mutants has verified a role for RecA in recombination since allele replacements can no longer be obtained in a *recA* background [19]. *B*. *burgdorferi recA* mutants do not show significantly increased sensitivity to DNA damage and neither do mutants in an additional 21 replication, recombination and repair genes, excepting functions of the nucleotide excision repair pathway [21,22]. Accordingly, *B*. *burgdorferi* are very sensitive to DNA damage. By becoming obligate parasites in tick and vertebrate hosts, *B*. *burgdorferi* may have evolved in a stable enough environment to dispense with the robust DNA repair capabilities of organisms with a RecA-modulated SOS response pathway [17].

A puzzling phenotype of *B*. *burgdorferi recA* mutants was the lack of effect on growth parameters, viability or a significant role in infectivity [19]. This implied that *B*. *burgdorferi* RecA plays no role in resetting paused or collapsed replication forks, that such replication issues rarely occur or that there may be an alternative RecA-like function that can provide these functions in the absence of functional RecA. *B*. *burgdorferi* maintain long-term infections, in part, by evasion of the adaptive immune system powered by an antigenic variation system that copies silent information from partial gene cassettes into an expression site for the *vlsE* lipoprotein gene [23,24]. Surprisingly, *vlsE* switching was found to be independent of RecA, despite a strong dependence of switching on the Holliday junction branch migrase, RuvAB [19,20,22]. The RuvAB involvement implied the creation of heteroduplex DNA in *vlsE* switching, a role normally played by the RecA recombinase. Again, this unexpected phenotype indicated that *B*. *burgdorferi* RecA may not play this expected role *in vivo*, or that there may be a redundant or specialized strand exchange function present.

In the present study, we present a purification and characterization of recombinant *B*. *burgdorferi* RecA to assess its biochemical properties and to address whether the unexpected phenotype of *recA* mutation may be related to unusual intrinsic properties of the protein. We show that *B*. *burgdorferi* RecA possesses many of the expected properties of bacterial RecA’s but find that DNA pairing reactions to be unusually dependent upon the presence of SSB. Additionally, we show that *B*. *burgdorferi* RecA is very sensitive to order-of-addition effects with SSB, indicating a probable need, *in vivo*, for recombinational mediator accessory functions. Finally, we show that long-range strand exchange reactions promoted by *B*. *burgdorferi* RecA to be slow. We conclude that the unusual properties of *recA* mutation in *B*. *burgdorferi* are not explained by any unusual, intrinsic biochemical property of its RecA protein.

## Materials and methods

### DNAs

ϕX174 virion and RFI DNAs were purchased from New England Biolabs (NEB). All oligonucleotides were purchased from Integrated DNA Technologies (IDT). Details of the oligonucleotides and substrate annealings used in this study are presented in the Supplementary information (S1 Table). Chromosomally encoded RecA from *B*. *burgdorferi* B31 (locus BB_0131) was synthesized as a reading frame codon-optimized for expression in Escherichia coli K12 and blunt-end cloned into pIDTSMART-AMP and verified by DNA sequencing (IDT). This plasmid was used for subsequent cloning into pET15b for expression in *E*. *coli*. The sequence of the codon-optimized RecA reading frame used in this study is presented in the Supplementary information as S1 Sequence.

### Proteins

*B*. *burgdorferi* RecA (locus BB_0131) and *B*. *burgdorferi* RecA (K88R) were purified as reported below. RecA (R88K) was generated by site-directed mutagenesis using the mutagenic oligonucleotides OGCB714/15 detailed in the Supplementary information (S1 Table). *B*. *burgdorferi* SSB (locus BB_0114) was purified as reported in [16]. *E*. *coli* LexA was purified as reported in the S1 Protocol. *E*. *coli* RecA was purchased from New England Biolabs as an N-terminal His-tagged version (RecA_f_) and *E*. *coli* SSB was purchased from Promega. BamHI, NdeI, XhoI, and T4 polynucleotide kinase were all purchased from New England Biolabs.

### Purification of RecA

A RecA expression strain in the BL21-based ‘rosetta’ strain was cultured at 37°C in LB media supplemented with 100 μg/mL ampicillin and 30 μg/mL chloramphenical to OD600 of 0.400. The culture was shifted to 24°C for 20 min and protein expression was induced by addition of IPTG to a final concentration of 0.25 mM. Incubation was continued at 24°C overnight. The culture was pelleted by centrifugation at 4000g for 15 minutes at 4.0°C. The pellet was resuspended in 25 mM HEPES (pH 7.6) 0.2 mM EDTA (pH 8.0) and 0.8 M KCl along with the protease inhibitor pepstatin (0.27 mM). The freeze-thaw method of cell lysis was employed. Lysozyme was added to 0.5 μg/ml and the cells were frozen at -80°C for one hour, thawed and refrozen three additional times. The lysate was produced by centrifugation at 10,000 g at 4°C for 90 minutes. The lysate was collected and NaCl was added to a final concentration of 1M. Samples were loaded to a 30 mL Ni-NTA column equilibrated with buffer containing (50 mM NaH2PO4, 10 mM imidazole, 10% glycerol, 1.0 M NaCl). The column was washed with 10 column volumes of wash buffer (50 mM NaH_2_PO_4_, 20 mM imidazole, 10% glycerol, 1.0 M NaCl) and eluted with 2 column volumes of elution buffer (50 mM NaH_2_PO_4_, 500 mM NaCl, 250 mM imidazole, 10% glycerol, 1.0 M NaCl). 20 μL aliquots were taken to examine the purification process and were run on 5/12% SDS-PAGE gels to identify peak fractions. The peak Ni-NTA fractions were further purified on a 10 mL hydroxylapatite (HAP) column equilibrated with HG 0.25M NaCl buffer (25 mM HEPES (7.6), 0.1 mM EDTA, 10% glycerol, 250 mM NaCl). The Ni-NTA peak fractions were pooled and the salt concentration adjusted to 0.25 M NaCl by dilution with HG buffer lacking salt prior to application to the HAP column. The HAP column was washed with 5 column volumes of HG 0.25M NaCl and with 1 column volume of HG 0.25 M NaCl + 0.2M NaPi (pH 7.6). RecA was eluted with 2 column volumes of HG 0.25M NaCl + 0.4M NaPi (pH 7.6). Aliquots were taken to examine the purification process by SDS-PAGE. Elution fractions were examined for the presence of ssDNA nuclease contamination tested at 37°C for 15 min in incubations using ~1 μM RecA. Nuclease-free peak fractions were pooled and dialyzed against 2 changes of 2L of buffer HG with 0.6 M NaCl for long-term storage of RecA at -80°C.

### DNA binding assays

Binding reactions were performed in a buffer containing 25 mM HEPES (pH 7.6), 2 mM MgCl_2_, 1 mM DTT, 50 mM NaCl and, where indicated, 2 mM ATP. The indicated concentrations of RecA were incubated for 5 min at 30°C with 5’-endlabeled substrate DNA (present at 950 nM nucleotides or basepairs for ssDNA and dsDNA, respectively). DNA binding was stabilized by protein-protein crosslinking induced by the addition of glutaraldehyde to a final concentration of 0.033%. Protein crosslinking was allowed to proceed at room temperature for 5 min. The samples were prepared for gel loading by addition of 5X load dye to a 1X concentration. 5X load dye contains 200 mM EDTA (pH 8), 32% glycerol, and 0.024% bromophenol blue. The samples were applied to 10 cm x 10 cm 6% PAGE 0.5X TBE gels run at 15V/cm in the cold room. The ssDNA substrate was 5’-endlabeled oligonucleotide OGCB396 while the duplex substrate was 5’-endlabeled oligonucleotide OGCB396 annealed to its complement OGCB742.

### ATP photoaffinity binding assay

The ability to bind ATP was assayed by incubating 2 μM RecA in reaction buffer containing 25 mM HEPES (pH 7.6), 2 mM MgCl_2_, 1 mM DTT, 50 mM NaCl, 25 μM ATP and 66 nM [γ^32^P]ATP in a total volume of 60 μL. After incubation on ice for 5 min the 1.5 mL reaction tubes were placed on a transilluminator and subjected to UV irradiation (312 nm) for the indicated times. Where indicated, ϕX174 virion DNA was present at 10 μg/ml. The samples were prepared for gel loading by addition of 5X SDS-load dye to a 1X concentration, followed by denaturation of the samples at 95°C for 6 min. After the gel run the gel was washed for 30 min in several changes of water then Coomassie stained to visualize the position of the bands. The gel was then wrapped in plastic and exposed to a phosphorimaging screen to detect protein that had become crosslinked to [γ^32^P]ATP.

### ATPase assays

ATPase activity was determined by incubating 1 μM RecA in buffer containing 25 mM HEPES (pH 7.6), 2 mM MgCl_2_, 1 mM DTT, 100 μg/ml bovine serum, 50 mM NaCl, 100 μM ATP and 66 nM [γ^32^P]ATP in a total volume of 30 μL. After incubation at 37°C for the times indicated in the figure legend, an aliquot of the reaction was spotted onto a polyethyleneimine thin-layer chromatography plate that was developed with 1M formic acid and 0.5M LiCl. The plate was air dried and then exposed to a phosphorimaging screen for determination of the free phosphate to total ATP ratio. When present, the indicated DNA effectors were present at 10 μg/mL.

### Strand invasion assays

The ability to form D-loops was assayed in a buffer containing 25 mM HEPES (pH 7.6), 2 mM MgCl_2_, 1 mM DTT, 100 μg/ml BSA, 50 mM NaCl and 2 mM ATP or AMPPNP. The reactions were performed in 120 μL reaction volumes by incubating 2 μM RecA with 20 nM of 5’-^32^P endlabeled 95 nt OGCB396 oligonucleotide (1.9 μM nt) at 30°C for 5 minutes followed by addition of pUC19 (77 μM nt) and incubation at 37°C for the indicated times. Where indicated, *B*. *burgdorferi* SSB was added to the indicated concentration. When added first, SSB was pre-incubated with the OGCB396 donor at 30°C for 5 min prior to RecA and pUC19 addition. When added second, SSB was added immediately before pUC19 addition. Reaction timepoints were examined by withdrawing 28 μL aliquots into 5X reaction stop dye to a final concentration of 1X. The 5X stop dye contains 100 mM Tris-HCl (pH 7.6), 10 mM MgCl_2_, 3% SDS, 30% glycerol, 0.024% bromophenol blue. Samples were deproteinated by addition of pronase to a final concentration of 50 μg/mL and by continued incubation at 37°C for 10 min. D-loops were visualized by loading the deproteinated samples on a 0.8% agarose 1X TAE gel electrophoresed at 3V/cm. The gel was transferred onto a Hybond N membrane on a 3MM Whatman paper backing and dried at 80°C under vacuum for 45 min prior to exposure to a phosphorimaging screen.

### Oligonucleotide-based strand exchange assays

The ability to perform strand exchange was assayed in a buffer containing 25 mM HEPES (pH 7.6), 2 mM MgCl_2_, 1 mM DTT, 100 μg/ml BSA, 50 mM NaCl and 2 mM ATP, AMPPNP or ATP[γS]. The reactions were performed in 60 μL reaction volumes by incubating 2 μM RecA with 20 nM of the 70 nt OGCB748 oligonucleotide (1.4 μM nt) at 30°C for 5 minutes followed by addition of 20 nM of the 5’-endlabeled 35 bp duplex assembled from oligonucleotides OGCB664/665 (1.4 μM nt) and continued incubation at 37°C for the indicated timepoints. Where present, SSB was added either just prior to addition of the duplex DNA substrate (added 2nd) or pre-incubated at 30°C for 5 min with the single-stranded donor DNA followed by RecA and duplex DNA addition and continued incubation at 37°C. Reaction timepoints were examined by withdrawing 18 μL aliquots into 5X reaction stop dye to a final concentration of 1X. The 5X stop dye contains 100 mM Tris-HCl (pH 7.6), 10 mM MgCl2, 3% SDS, 30% glycerol, 0.024% bromophenol blue. Samples were deproteinated by addition of pronase to a final concentration of 50 μg/mL followed by continued incubation at 37°C for 10 min. The strand exchange reaction timecoureses were visualized by loading the deproteinated samples on an 8% PAGE 1X TAE/0.1% SDS gel electrophoresed at 10V/cm. The gel was transferred to 3MM Whatman paper and dried at 80°C under vacuum prior to exposure to a phosphorimaging screen.

### ϕX174 strand exchange assays

The ability to perform strand exchange with long substrates was assayed in a buffer containing 25 mM HEPES (pH 7.6), 1 mM DTT, 100 μg/ml BSA, 50 mM NaCl and 2 mM ATP or ATP[γS] and the concentration of MgCl_2_ indicated in the legends. The reactions were performed in 120 μL reaction volumes by incubating 2 μM RecA with ϕX174 virion (5.1 μM nt) at 30°C for 5 minutes followed by addition of XhoI-linearized, ϕX174 RF1 DNA (5.1 μM nt) followed by continued incubation at 37°C for the indicated timepoints. When added first, SSB was incubated with ϕX174 virion at 30°C for 5 min prior to the addition of RecA and linear ϕX174 duplex DNA followed by continued incubation at 37°C for the times indicated above the gel. When SSB was added second, RecA was incubated with ϕX174 virion at 30°C for 5 min prior to the addition of SSB and linear duplex DNA, followed by continued incubation at 37°C for the times indicated above the gel. Reaction timepoints were examined by withdrawing 18 μL aliquots into 5X reaction stop dye to a final concentration of 1X. The 5X stop dye contains 100 mM Tris-HCl (pH 7.6), 10 mM MgCl2, 3% SDS, 30% glycerol, 0.024% bromophenol blue. Samples were deproteinated by addition of pronase to a final concentration of 50 μg/mL followed by continued incubation at 37°C for 30 min. Strand exchange products were visualized by loading the deproteinated samples to a 0.8% agarose 1X TAE gel electrophoresed at 3V/cm for 4 h. The gel was transferred to DE81 paper and dried at 80°C under vacuum for 45 min prior to exposure to a phosphorimaging screen.

### Coprotease assays

*B*. *burgdorferi* RecA was assayed for coprotease activity with *E*. *coli* LexA. 60 μL reactions containing 25 mM HEPES (pH 8.2), 1 mM MgCl_2_, 1 mM DTT, 50 mM NaCl and, where indicated 1 mM ATPγS or ATP. In reactions with ssDNA 2 μM (nt) of OGCB396 was pre-incubated with 1.25 μM RecA at 30°C for 5 min prior to addition of 1.5 μM LexA and continuation of the incubation at 30°C for 16 h. Control reactions without RecA were conducted as above without inclusion of RecA. LexA autocleavage was visualized by denaturation of the samples in SDS/DTT load dye and application to a 5/18% SDS-PAGE gel. The gel was stained with Coomassie Brilliant Blue. Reactions supplemented with *B*. *burgdorferi* SSB contained 190 nM SSB that was added just before LexA. This gel was Coomassie stained and then silver stained to improve detection of the low abundance SSB.

## Results and discussion

### *B*. *burgdorferi* RecA displays preferential, ATP-stimulated affinity for ssDNA

**Fig 1:**
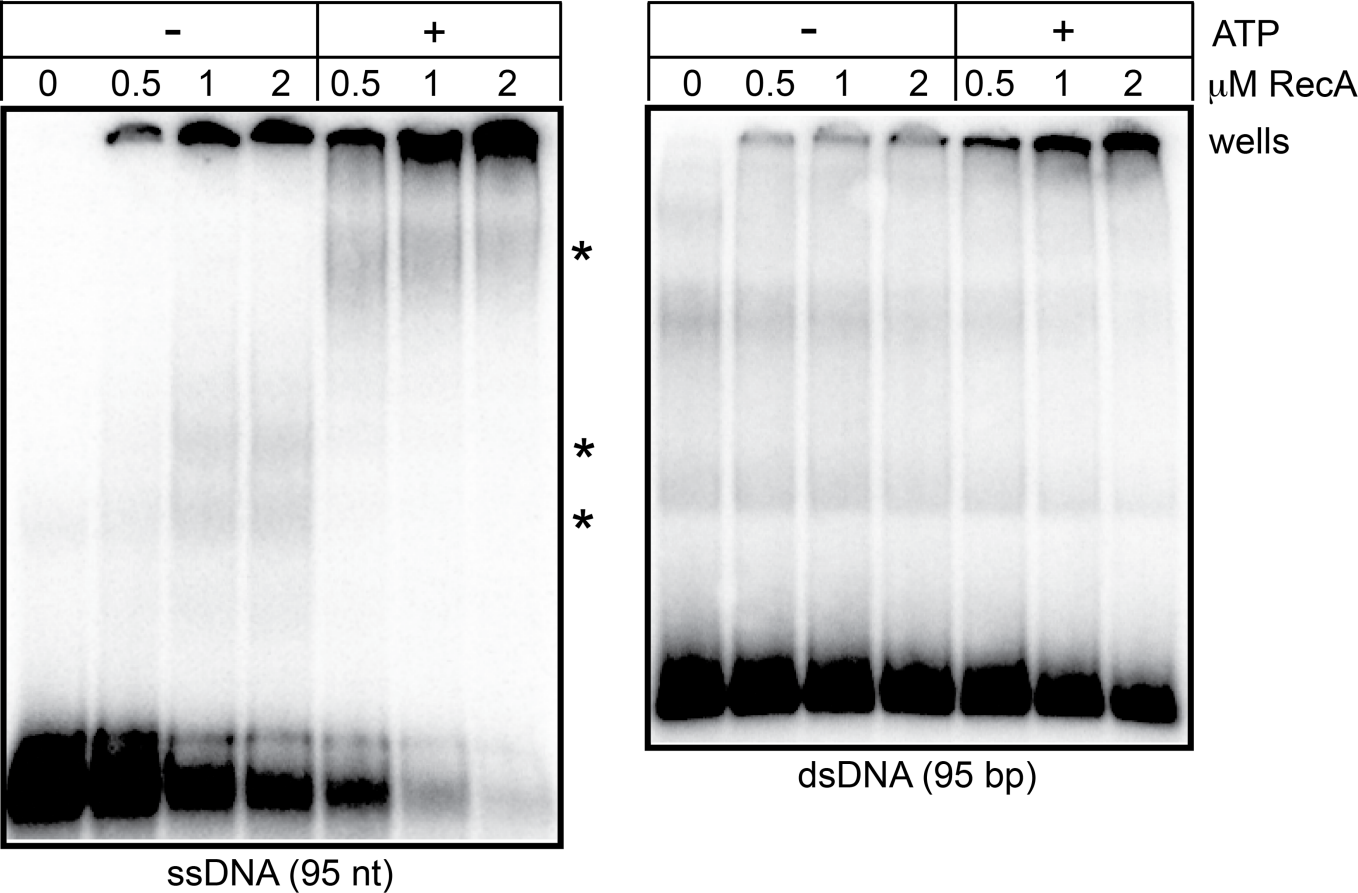
*B*. *burgdorferi* RecA binds preferentially to ssDNA. 6% PAGE 0.5X TBE gel analysis of RecA binding to 95 nt ssDNA and 95 bp dsDNA. The indicated concentrations of RecA were assayed for binding with 10 nM (molecules) of the indicated 5-endlabeled DNAs in a buffer containing 25 mM HEPES (pH 7.6), 2 mM MgCl_2_, 1 mM DTT, 50 mM NaCl and, where indicated, 2 mM ATP, by incubation at 30°C for 5 min. This was followed by protein crosslinking with glutaraldehyde (final concentration 0.033%) at 20°C for 5 min prior to application of the binding reactions to native a PAGE gel. The gel migration position of RecA-DNA complexes is indicated by asterisks and the position of the wells is noted. See the Materials and methods section for details.

Recombinase binding to single-stranded DNA is required for assembly of the presynaptic filament that conducts all the catalytic steps of recombination. Different mechanisms are used to load various recombinases to the ssDNA substrate. *E*. *coli* RecA has an intrinsic, kinetic preference for binding ssDNA over duplex DNA and this intrinsic, high affinity state for ssDNA is stabilized by ATP binding [25]. An intrinsic preference for ssDNA prevents titration of RecA *in vivo* by the vast excess of duplex DNA present. We assayed *B*. *burgdorferi* RecA for its intrinsic affinity for ssDNA vs. dsDNA by an electrophoretic mobility shift assay coupled with protein-protein crosslinking to stabilize RecA multimers (Fig 1; [26]). *B*. *burgdorferi* RecA displayed the expected intrinsic preference for ssDNA over duplex DNA. This affinity increases with addition of ATP to the binding buffer. ATP binding also results in a single, discrete higher molecular weight bandshift and well shifts, probably indicative of continuous presynpatic filament formation trapped by the protein-protein crosslinking performed before gel loading (Fig 1).

### *B*. *burgdorferi* RecA displays DNA-dependent ATPase activity preferentially stimulated by ssDNA

**Fig 2:**
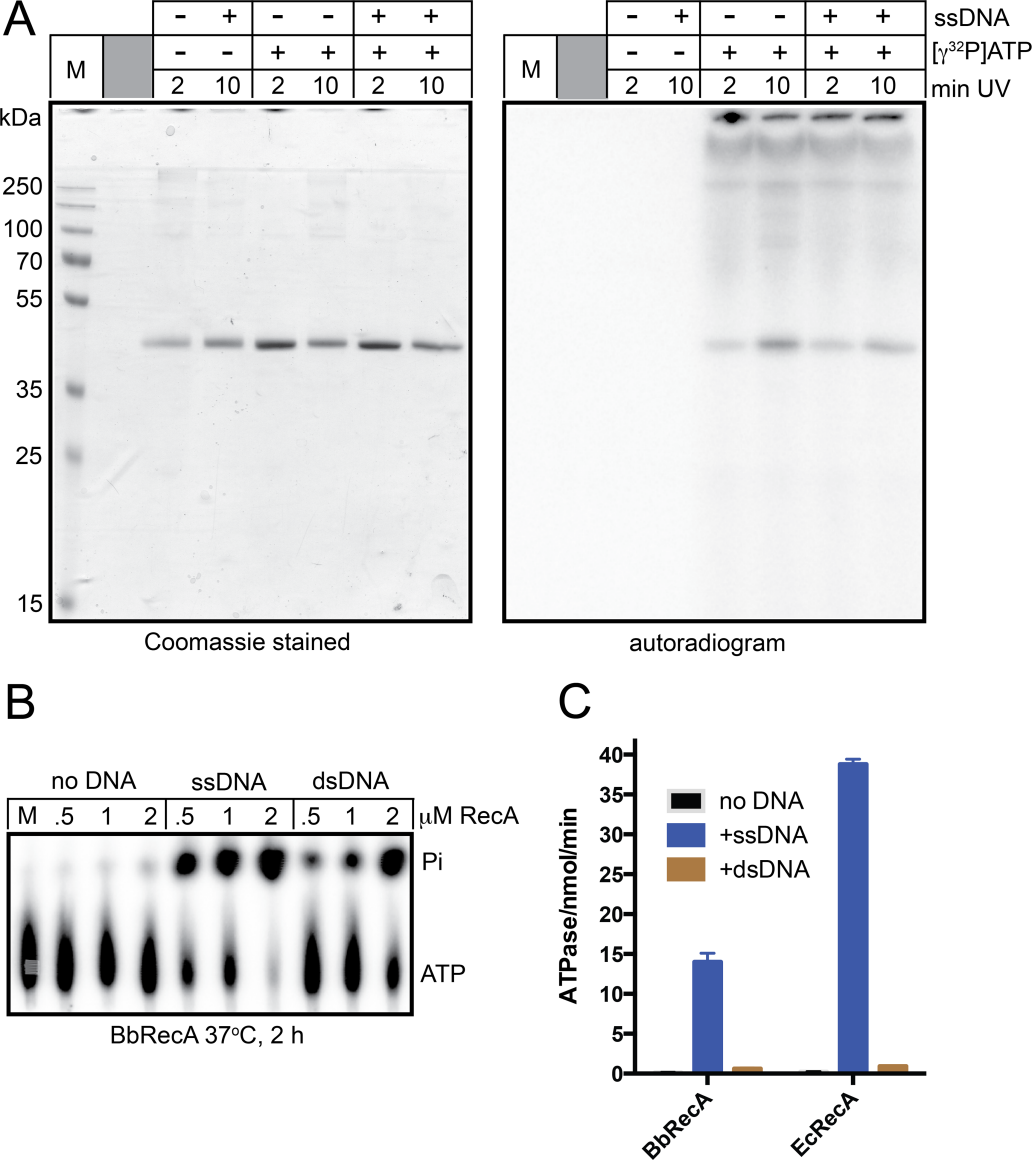
*B*. *burgdorferi* RecA binds ATP and possesses DNA-dependent ATPase activity. A) 12% SDS-PAGE analysis of RecA ATP photoaffinity binding assays. ATP binding was assayed by incubating 2 μM RecA in reaction buffer containing 25 mM HEPES (pH 7.6), 2 mM MgCl_2_, 1 mM DTT, 50 mM NaCl, 25 μM ATP and 66 nM [γ^32^P]ATP for 5 min on ice followed by photocrosslinking (312 nm) for the times indicated above the gel. Where indicated, ϕX174 virion DNA was present at 10 μg/ml. M denotes the protein molecular weight marker. B) Polyethyleneimine thin-layer chromatography ATPase assay results with the indicated concentrations of RecA -/+ 10 μg/mL ϕX174 virion (ssDNA) and ϕX174 RF1 (dsDNA). ATPase activity was assayed by incubating the indicated concentrations of RecA in buffer containing 25 mM HEPES (pH 7.6), 2 mM MgCl_2_, 1 mM DTT, 100 μg/ml bovine serum, 50 mM NaCl, 100 μM ATP and 66 nM [γ^32^P]ATP in a total volume of 30 μL for 2 hours. M denotes a 2 hour mock incubation of [γ^32^P]ATP without added DNA or RecA. C) Summary of ATPase assay results with *B*. *burgdorferi vs*. *E*. *coli* RecA, -/+ 10 μg/mL ϕX174 virion or ϕX174 RF1 DNA. ATPase assays containing 1 μM RecA were incubated at 37°C for 5 min for ϕX174 virion (ssDNA) containing reactions or at 37°C for 120 min for DNA-free and ϕX174 RF1 (dsDNA) containing reactions. The mean and standard deviation of at least three independent experiments is shown.

Presynaptic recombinase filaments are dynamic structures that polymerize with a polarity preference by adding new ATP bound protomers preferentially at one end and age, or undergo turnover by ATP hydrolysis at the other end of the filament. All RecA-like proteins are DNA-dependent ATPases, though there is a wide range of ATP hydrolysis rates that correlates with the size and complexity of the genome harbouring the recombinase [6]. T4 UvsX has the fastest rate, 20 times faster than *E*. *coli* RecA which is, in turn, 15 times faster than yeast Rad51 [6]. We assessed *B*. *burgdorferi* RecA’s ability to bind ATP and compared its DNA-dependent ATPase activity with that of *E*. *coli* RecA (Fig 2). We found, by an ATP photoaffinity crosslinking approach, that *B*. *burgdorferi* RecA binds ATP (Fig 2A). We also found that it possesses DNA-dependent ATPase activity preferentially stimulated by ssDNA (Fig 2B & 2C). The ssDNA-stimulated rate of ATP hydrolysis is 2.8-fold slower than that possessed by *E*. *coli* RecA assayed under identical conditions (Fig 2C). Conserved Walker A and Walker B motifs are important determinants of ATP binding and hydrolysis in RecA orthologues. *B*. *burgdorferi* RecA is 54% identical and 76% similar in sequence alignments with *E*. *coli* RecA, possessing conserved Walker A and B motifs (S1 Fig). *B*. *burgdorferi* and *E*. *coli* SSBs are much more divergent (S2 Fig). We constructed an analogue of the well-characterized Walker A mutant of *E*. *coli* RecA (K72R) to make *B*. *burgdorferi* RecA (K88R). *E*. *coli* RecA (K72R) binds ATP but hydrolyses it very slowly [27]. *B*. *burgdorferi* RecA (K88R) was predicted to have similar properties. ATP binding and hydrolysis assays for this mutant showed that the K88R mutant could bind ATP and showed an impaired DNA-dependent ATP hydrolysis rate, though the rate reduction is only 4.8-fold relative to wild type rather than the 600-fold reduction reported for *E*. *coli* RecA (K72R) (S3 Fig; [27]).

### *B*. *burgdorferi* RecA-promoted strand invasion is strongly dependent upon SSB but very sensitive to the order of SSB addition

**Fig 3:**
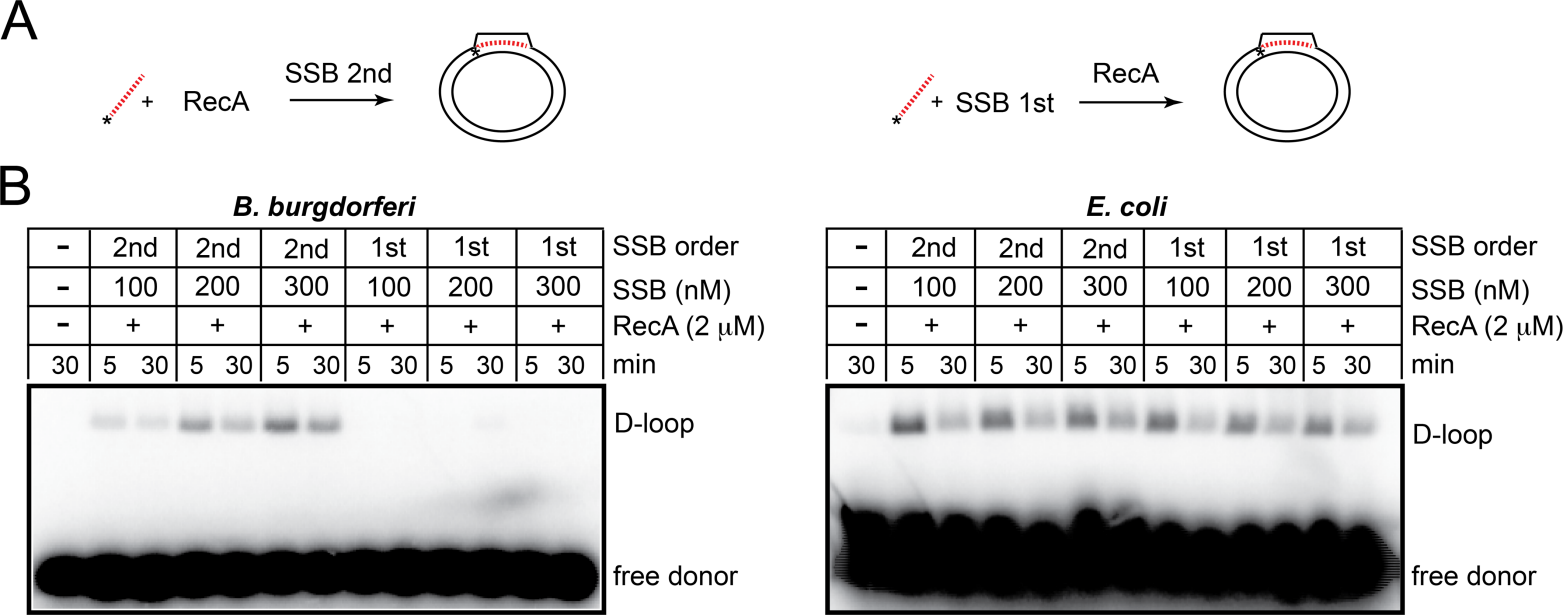
*B*. *burgdorferi* RecA-promoted strand invasion is strongly dependent upon SSB but sensitive to the order of SSB addition. A) Schematic representation of the strand invasion assay indicating the effect of SSB order-of-addition on D-loop recovery. The 95 nt 5’-endlabeled ssDNA donor and supercoiled pUC19 target DNA are depicted. B) 0.8% agarose 1X TAE gel analysis of D-loop formation in reactions supplemented with a titration of SSB added either after RecA-ssDNA pre-incubation (SSB 2^nd^) or pre-incubated with the ssDNA donor before RecA and pUC19 addition (SSB 1^st^). D-loop formation was assayed in a buffer containing 25 mM HEPES (pH 7.6), 2 mM MgCl_2_, 1 mM DTT, 100 μg/ml BSA, 50 mM NaCl and 2 mM ATP or AMPPNP in a 120 μL reaction volume. The assay was conducted as a staged reaction with pre-incubation of RecA or SSB with the donor ssDNA (30°C, 5 min) followed by addition of SSB or RecA and supercoiled pUC19 target plasmid and continued incubation at 37°C for the indicated times. RecA was present at 2 μM, 5’-^32^P endlabeled 95 nt donor ssDNA at 1.9 μM (nt) and pUC19 at 77 μM (nt). Reaction panels using *B*. *burgdorferi* and *E*. *coli* RecA and SSB are shown. See Materials and methods for additional details.

**Fig 4:**
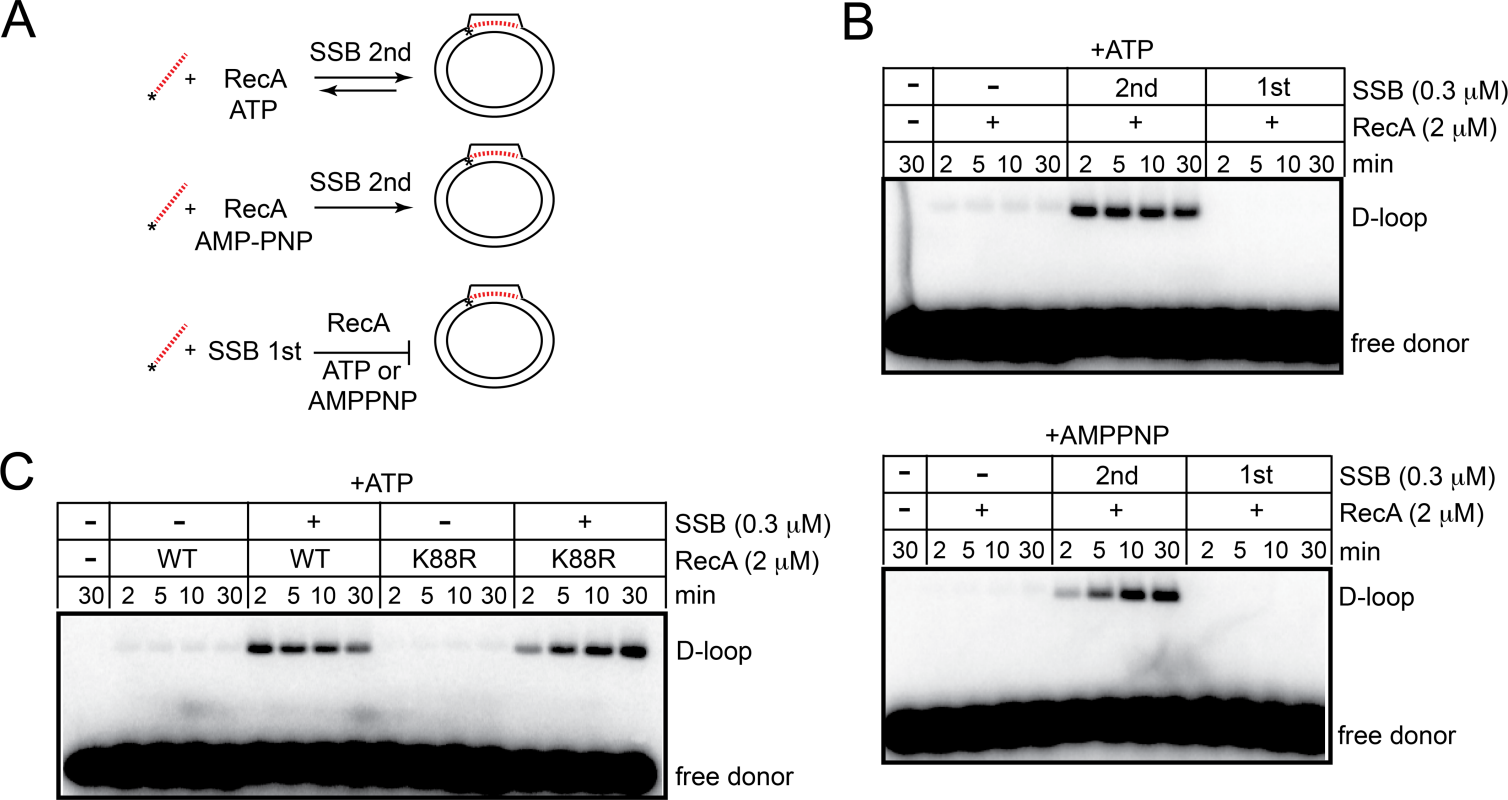
Strand invasion is highly reversible when RecA can hydrolyze ATP. A) Schematic representation of the strand invasion assay indicating the effect of SSB order of addition and ATP hydrolysis on D-loop recovery. B) 0.8% agarose 1X TAE gel analysis of strand invasion timecourses performed -/+ 0.3 μM SSB addition (added 2^nd^ vs. added 1^st^) in buffer containing ATP (top panel) or AMPPNP (bottom panel). Reaction conditions were as described in the legend for Fig 3 and in the Materials and methods section. C) 0.8% agarose 1X TAE gel analysis of strand invasion timecourses performed -/+ 0.3 μM SSB addition (added 2^nd^) comparing the behavior of wild type RecA *vs*. the Walker A mutant, RecA (K88R) in a buffer containing ATP.

The RecA nucleoprotein presynaptic filament performs the homology search and stably invades homologous duplex DNA forming gel-stable D-loop structures. D-loop formation depends upon a ssDNA end and negative supercoiling in the homologous duplex target DNA [28]. Some RecA orthologues, such as *E*. *coli* RecA, can form stable D-loops without accessory factors while others such as yeast Rad51 have a strong dependence upon single-stranded DNA binding protein (RPA). Additionally, a DNA translocase, Rad54, strongly stimulates strand invasion [29,30,31,32]. Strand invasion assays performed with *B*. *burgdorferi* RecA showed an extremely weak strand invasion activity unless the reaction was supplemented with SSB (Figs 3 & 4). *E*. *coli* RecA, assayed under identical buffer conditions, does not show a similar dependence upon its cognate SSB (Fig 3B; right panel). When SSB is allowed to pre-bind the ssDNA, strand invasion by *B*. *burgdorferi* RecA is completely blocked (Fig 3B; left panel). *E*. *coli* RecA was able to displace its cognate SSB and form D-loops, even when SSB was present at a saturating concentration (300 nM). *B*. *burgdorferi* RecA’s extreme sensitivity to this SSB order-of-addition effect and its strong dependence upon SSB is reminiscent of *Streptococcus pneumoniae* RecA’s complete inhibition by SSB pre-binding to ssDNA and by the effect of RPA on Rad51 [29,33,34]. *In vivo*, RecA will often need to be loaded to ssDNA prebound by SSB. *B*. *burgdorferi* RecA’s inability to displace its cognate SSB from ssDNA implies a strong need for a mediator activity to promote SSB/RecA exchange [6]. Interestingly, the *B*. *burgdorferi* genome lacks homologues of the RecFOR proteins that normally play this role in eubacteria, implying that non-canonical mediator functions may exist [16,35].

Our strand invasion assay conditions have a relatively high NaCl concentration (50 mM) and a very low concentration of free Mg^2+^ (2 mM Mg^2+^/2 mM ATP). These conditions minimize secondary structure formation in the ssDNA substrate and allow us to infer that the stimulatory effect of SSB addition is likely primarily through postsynaptic stabilization of the displaced strand, preventing secondary invasion events or reversion back to substrates [14,33,36]. Many RecA orthologues are stimulated by SSBs at the pre-synaptic stage where removal of secondary structure in ssDNA aids complete presynaptic filament formation as well as at the postsynaptic stage by stabilization of the displaced strand [12,13,14].

### *B*. *burgdorferi* RecA-promoted strand invasion is dynamic in the presence of SSB and hydrolysable ATP

The results presented in Fig 4 indicated that strand invasion promoted by *B*. *burgdorferi* RecA and SSB is a dynamic process, the yield of D-loops peaked at early timepoints and decayed back to substrate DNAs as the incubation time was extended (Fig 4B, panel with ATP). This dynamic behaviour was not due to ATP depletion since the presence of an ATP regeneration system did not change the dynamic response and even reduced overall D-loop yield (S4 Fig). *E*. *coli* RecA also showed a similar response under identical buffer conditions (Fig 3B). Presynaptic filament dynamics are promoted by competition for binding to ssDNA from SSB, promoting a directional treadmilling of the filament as ADP-bound recombinbase at the 5’-end of the filament is displaced by SSB [37]. This likely stimulates the rapid but reversible strand invasion observed in Figs 3 & 4. When the non-hydrolysable ATP analogue, AMPPNP, was used a gradual, time-dependent increase in D-loop yield resulted (Fig 4B, panel with AMPPNP). AMPPNP stabilizes RecA/DNA filaments. Here, the stimulatory effect of SSB on strand invasion can be mostly attributed to a postsynaptic role in stabilizing the displaced strand, since a role in RecA-ssDNA filament turnover and reinvasion can be ruled out. To ensure that the stabilization of the RecA filament was not specific to AMPPNP we tested the behaviour of the RecA (K88R) mutant in the strand invasion assay (Fig 4C). A similar stabilizing effect was seen with ATP when the Walker A mutant RecA (K88R) was used to allow ATP binding but to inhibit ATP hydrolysis. The time-dependent accumulation of D-loops with RecA (K88R) phenocopied the behaviour of wild type RecA used with AMPPNP (Fig 4C).

Since SSB pre-binding to the single-stranded donor DNA strongly inhibited strand invasion by RecA it was of interest to determine which protein accessed the ssDNA first when both were present at the time of ssDNA addition. S5B Fig shows, despite RecA being present in 6.67-fold molar excess, when both proteins were present simultaneously that strand invasion was inhibited to the same degree as the condition where SSB was pre-bound to the ssDNA. This was true with hydrolysable ATP and with AMPPNP, indicating that SSB loads first and resists being exchanged by RecA.

### *B*. *burgdorferi* RecA-promoted strand exchange with short (oligonucleotide) substrates is strongly stimulated by SSB

**Fig 5:**
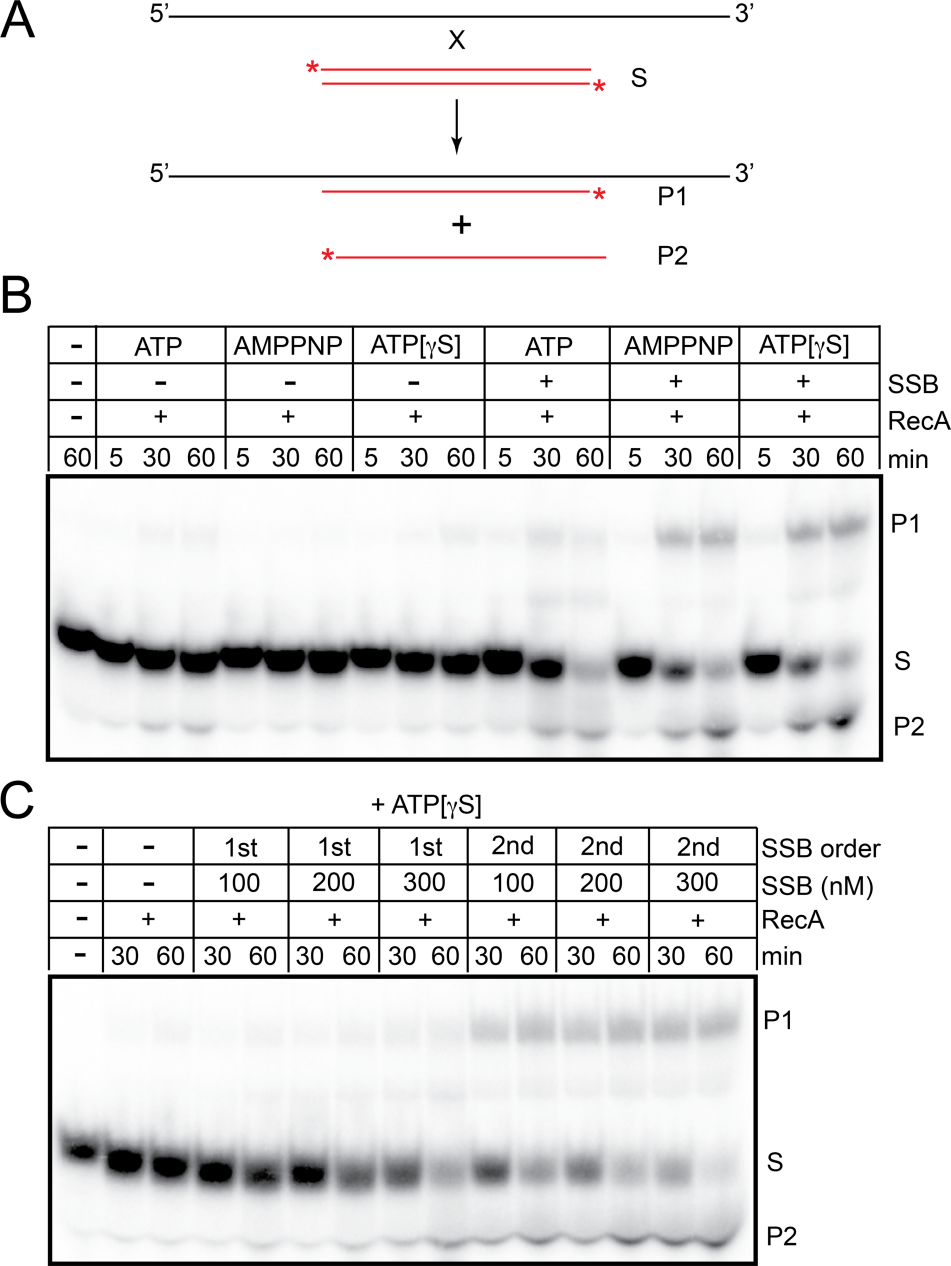
*B*. *burgdorferi* RecA-promoted strand exchange with oligonucleotide substrates is strongly stimulated by SSB. A) Schematic representation of the oligonucleotide substrates used. The ssDNA donor oligonucleotide is 70 nt in length while the 5’-endlabeled duplex target DNA is 35 bp. * denote the ^32^P-endlabels, red/shaded lines represent endlabeled strands.B) 8% PAGE 1X TAE/0.1% SDS gel analysis of strand exchange reactions with RecA, -/+ SSB. Strand exchange was assayed in a buffer containing 25 mM HEPES (pH 7.6), 2 mM MgCl_2_, 1 mM DTT, 100 μg/ml BSA, 50 mM NaCl and 2 mM ATP, AMPPNP or ATP[γS] in 60 μL reaction volumes. The assay was conducted as a staged reaction with pre-incubation of RecA with the donor ssDNA (30°C, 5 min) followed by addition of 5’-endlabeled duplex target DNA (S) and continued incubation at 37°C. RecA was present at 2 μM, donor ssDNA at 1.4 μM (nt) and 5’-endlabeled target duplex (S) was present at 1.4 μM (nt). When added, SSB was added after pre-incubation of RecA with the single stranded donor and was present at 300 nM. The proteins and nucleotide co-factor present are indicated in the loading key above the gels. C) 8% PAGE 1X TAE/0.1% SDS gel analysis of strand exchange reactions with RecA and a titration of SSB added before RecA (1st) or after RecA incubation with single-stranded donor DNA (2nd). Reactions were performed with ATP[γS] as the nucleotide co-factor.

Strand exchange with short oligonucleotide substrates promoted by *B*. *burgdorferi* RecA was also found to be dependent upon inclusion of SSB in the reaction, showing a similar order-of-addition effect to that seen in the strand invasion assays (Fig 5). *S*. *pneumoniae* RecA and the eukaryotic RecA orthologue, Rad51, show similar strong dependence of strand exchange on the presence of cognate single-stranded DNA binding proteins where they play a strong post-synaptic role in stabilizing DNA pairing [14,33,36]. However, where this has been studied with short substrates as in the case of Rad51, the stabilization of DNA pairing afforded by RPA was only required in reactions with plasmid length molecules not with oligonucleotide substrates [36]. Strand exchange reactions performed with non-hydrolysable ATP analogues were found to give the highest yield of strand exchange products (Fig 5B). The RecA (K88R) mutant used with ATP showed robust strand exchange in SSB supplemented conditions, providing independent verification that strand exchange with the oligonucleotide substrates was not dependent upon ATP hydrolysis (S6 Fig). *E*. *coli* RecA has previously been shown to be able to promote strand invasion and quite extensive 3-strand exchange by binding ATP without attendant ATP hydrolysis [38,39].

*E*. *coli* RecA used under identical buffer conditions promoted strand exchange but was not stimulated by addition of its cognate SSB (S7 Fig). The strand exchange reaction promoted by *E*. *coli* RecA also displayed the same preference for ATP[γS] over ATP that the *B*. *burgdorferi* enzyme displayed under these conditions (S7 Fig). This likely indicates that the relatively high salt reaction conditions used in the strand exchange assay favours conditions that stabilize RecA-DNA filaments and disfavours reaction reversal.

### *B*. *burgdorferi* RecA-promoted strand exchange with long substrates is dependent upon SSB and ATP hydrolysis

**Fig 6:**
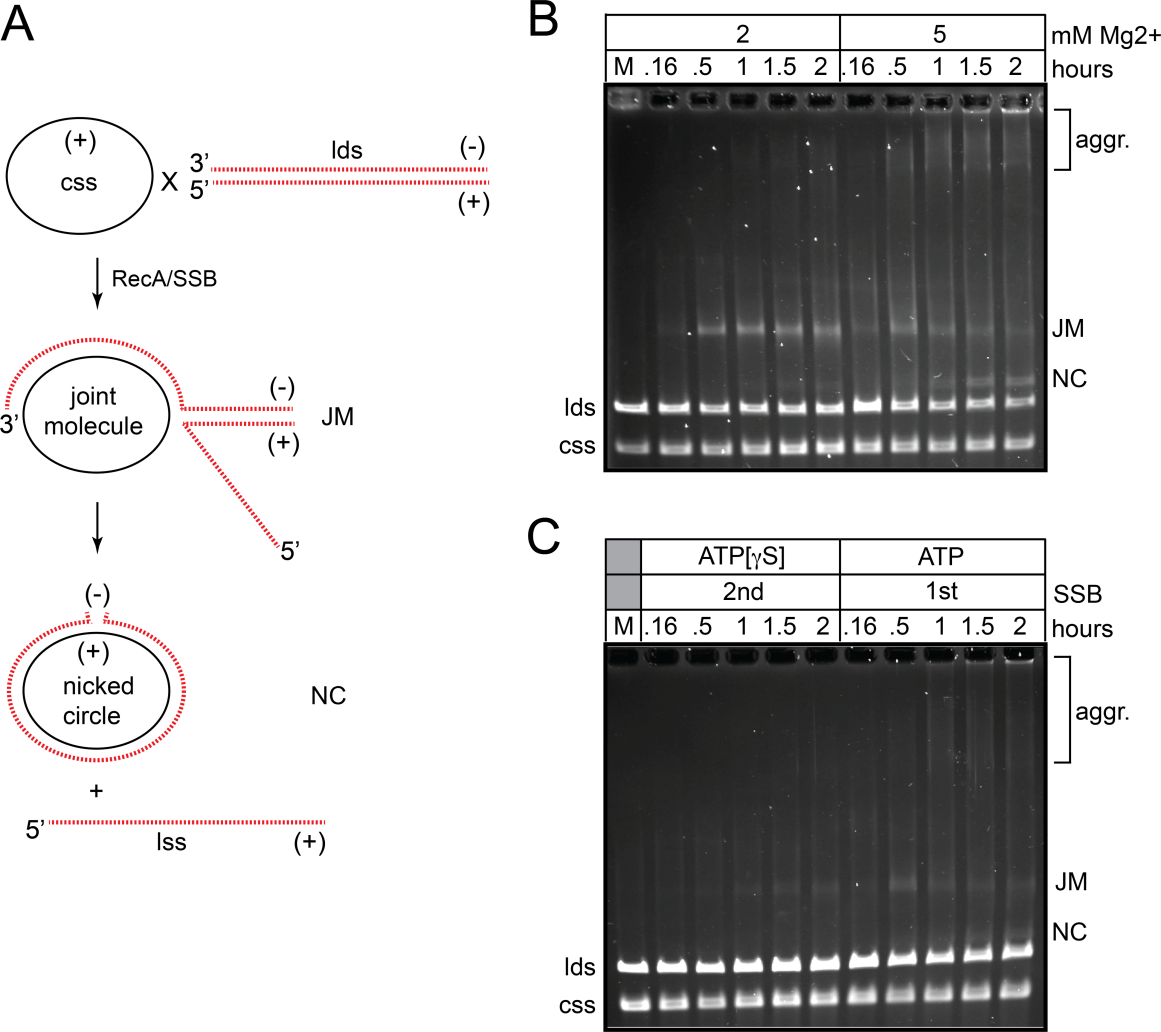
*B*. *burgdorferi* RecA-promoted strand exchange with long substrates. A) Schematic of the strand exchange assay using ϕX174 virion DNA (closed circular ssDNA; css) and XhoI-linearized ϕX174 duplex DNA (linear dsDNA; lds). As strand exchange initiates, joint molecule (JM) intermediates form. As strand exchange progresses, the final strand exchange products of nicked circular DNA (NC) and linear ssDNA (lss) are formed. Partial, intermolecular strand exchange between multiple donor and target molecules can also give rise to large aggregates (aggr.). B) 0.8% agarose 1X TAE gel analysis of strand exchange reactions performed with RecA and SSB. Strand exchange was assayed in a buffer containing 25 mM HEPES (pH 7.6), 1 mM DTT, 100 μg/ml BSA, 50 mM NaCl, 2 mM ATP and the indicated concentration of MgCl_2_ in 120 μL reaction volumes. The assay was conducted as a staged reaction with pre-incubation of RecA with the donor ssDNA (css; 30°C, 5 min) followed by addition of SSB and linear duplex DNA (lds) and continued incubation at 37°C. RecA was present at 2 μM, ϕX174 virion (css) at 5.1 μM (nt) and XhoI-linearized ϕX174 duplex DNA (lds) at 5.1 μM (nt). SSB was added after pre-incubation of RecA with the single stranded donor and was present at 0.9 μM. The concentration of added MgCl_2_ is noted in the loading key above the gel. The migration position of the substrate DNA is noted to the left of the gel and of the products to the right. Under our gel conditions css and lss have identical gel mobilities. M denotes mock incubation of the substrate DNAs without added RecA or SSB. C) 0.8% agarose 1X TAE gel analysis of SSB order-of-addition effects and ATP hydrolysis requirements of strand exchange reactions performed with long substrates. Strand exchange reactions were performed as noted in B) using buffer conditions with the optimal 5 mM MgCl_2_ concentration and 0.9 μM of added SSB. The order of SSB addition and nucleotide cofactor present is indicated in the loading key above the gel. When added first, SSB was incubated with ϕX174 virion at 30°C for 5 min prior to the addition of RecA and linear ϕX174 duplex DNA followed by continued incubation at 37°C for the times indicated above the gel. When SSB was added second, RecA was incubated with ϕX174 virion at 30°C for 5 min prior to the addition of SSB and linear duplex DNA, followed by continued incubation at 37°C for the times indicated above the gel. M denotes mock incubation of the substrate DNAs without added RecA or SSB.

**Fig 7:**
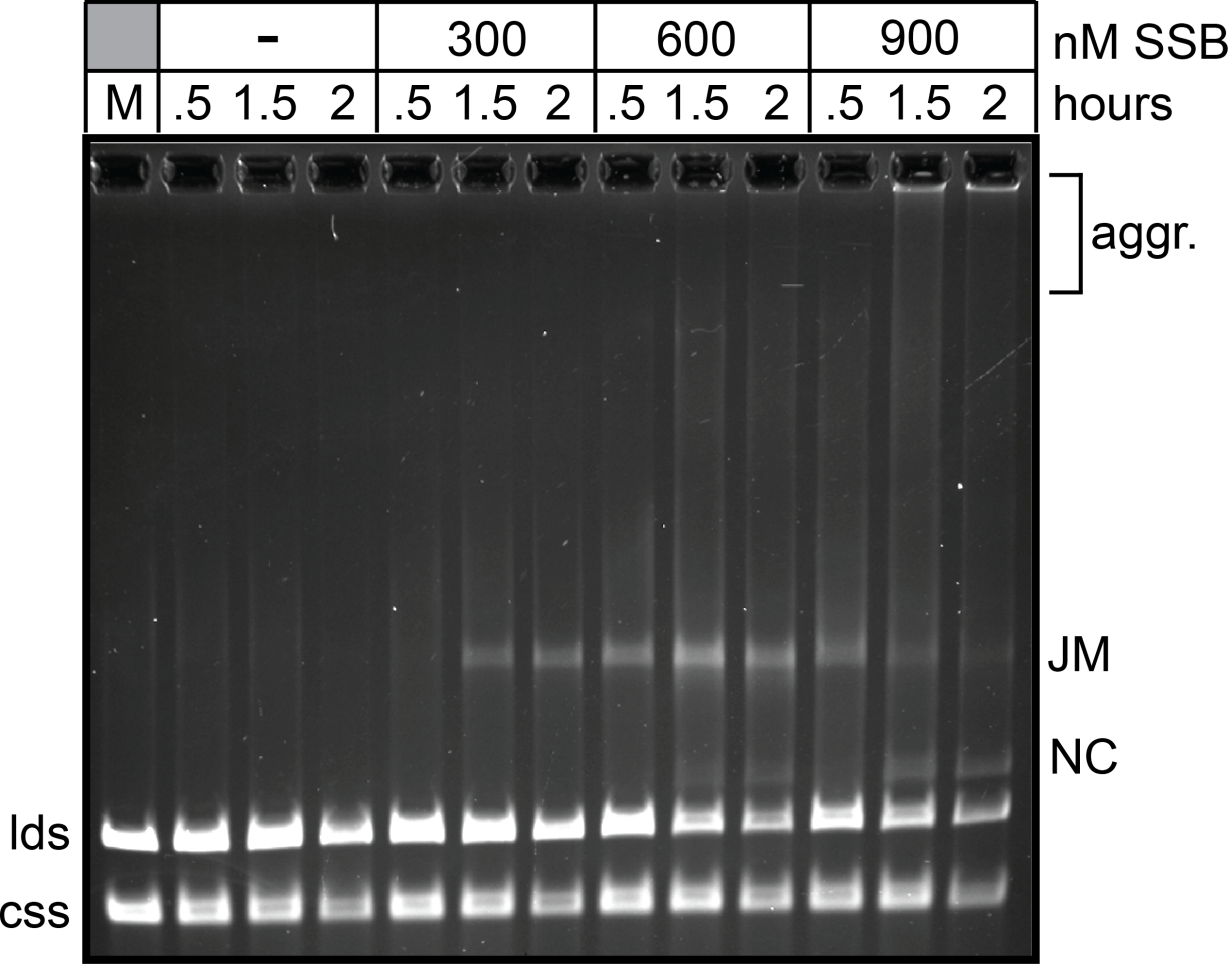
*B*. *burgdorferi* RecA-promoted strand exchange with long substrates is dependent upon SSB. 0.8% agarose 1X TAE gel analysis of strand exchange reactions performed with RecA and a titration of SSB in a buffer with hydrolysable ATP. RecA was present at 2 μM, ϕX174 virion (css) at 5.1 μM (nt) and XhoI-linearized ϕX174 duplex DNA (lds) at 5.1 μM (nt). When present, SSB was added after RecA pre-incubation with ϕX174 virion and was present at the concentration noted in the loading key above the gel. The gel labels are as described in the legend to Fig 6.

The ability of *B*. *burgdorferi* RecA to promote long-range strand exchange was tested in the standard 3-strand exchange assay using ϕX174 substrates (5386 nt; Figs 6 & 7). With long substrate molecules and buffer conditions developed for the strand invasion and strand exchange with short substrates *B*. *burgdorferi* RecA forms mostly joint molecules rather than full products of strand exchange in a 2-hour timecourse (Fig 6B; 2 mM Mg^2+^ panel). Bacterial RecA proteins are often sensitive to Mg^2+^ concentration in the 3-strand assay so we tested conditions where the concentration of free Mg^2+^ was elevated [40,41]. Using conditions with 2 mM ATP and 5 mM Mg^2+^ we observed conversion of the joint molecules (JM) into products that had undergone further strand exchange (Fig 6B; 5 mM Mg^2+^ panel). Reactions with 8 or 10 mM Mg^2+^ produced only joint molecules (data not shown). The full recombinant product of nicked circular (NC) DNA was produced as well as large molecular weight aggregates that are likely the result of partial, intermolecular strand exchange between multiple donor and target molecules. Compared to reactions with this system promoted by *E*. *coli* RecA/SSB with optimal buffer conditions for the *E*. *coli* proteins, the reaction promoted by *B*. *burgdorferi* RecA/SSB is slow, though doubtless adequate for an organism with a ~6 h doubling time [40]. The buffer conditions optimized for the *B*. *burgdorferi* proteins supports little reaction with the *E*. *coli* proteins (S8 Fig). As observed for strand invasion and strand exchange with short substrates, strand exchange with long substrates was inhibited when SSB was pre-incubated with ssDNA prior to RecA addition, though the inhibitory effect is not as strong as that seen with short substrates (Fig 6C; SSB 1^st^ panel). This again highlights the probable need *in vivo* for a recombinational mediator to load RecA to SSB-coated ssDNA. In contrast to strand exchange with short substrates, long range strand exchange required ATP hydrolysis, since a reaction with APT[γS] formed only joint molecules and and some aggregates rather than the full length recombinant product of nicked circular DNA (Fig 6C; SSB 2^st^ ATP[γS] panel). This is consistent with what is observed for bacterial RecA’s but contrasts with eukaryotic Rad51, which does not have a strong requirement for ATP hydrolysis in this assay [38,39,42].

The strand pairing activity of *B*. *burgdorferi* RecA was shown in Figs 3–5 to be strongly dependent upon the presence of SSB. We tested the requirement for SSB in the ϕX174-based 3-strand exchange assay by performing an SSB titration (Fig 7). The reactions possess the optimal 5 mM concentration of Mg^2+^ and, when added, SSB was added after pre-incubation of RecA with ssDNA. The reaction without SSB failed to yield products of any kind, while addition of 0.3 μM SSB supported slow formation of some joint molecules. Addition of 0.6 and 0.9 μM SSB supported progressively faster reactions that started to produce detectable levels of nicked circular strand exchange products in the 2-hour timecourse tested (Fig 7). As with DNA pairing assayed with short substrates, long-range DNA strand exchange by *B*. *burgdorferi* RecA is strongly dependent upon its cognate SSB.

### *B*. *burgdorferi* RecA possesses a weak DNA-independent coprotease activity with *E*. *coli* LexA

**Fig 8:**
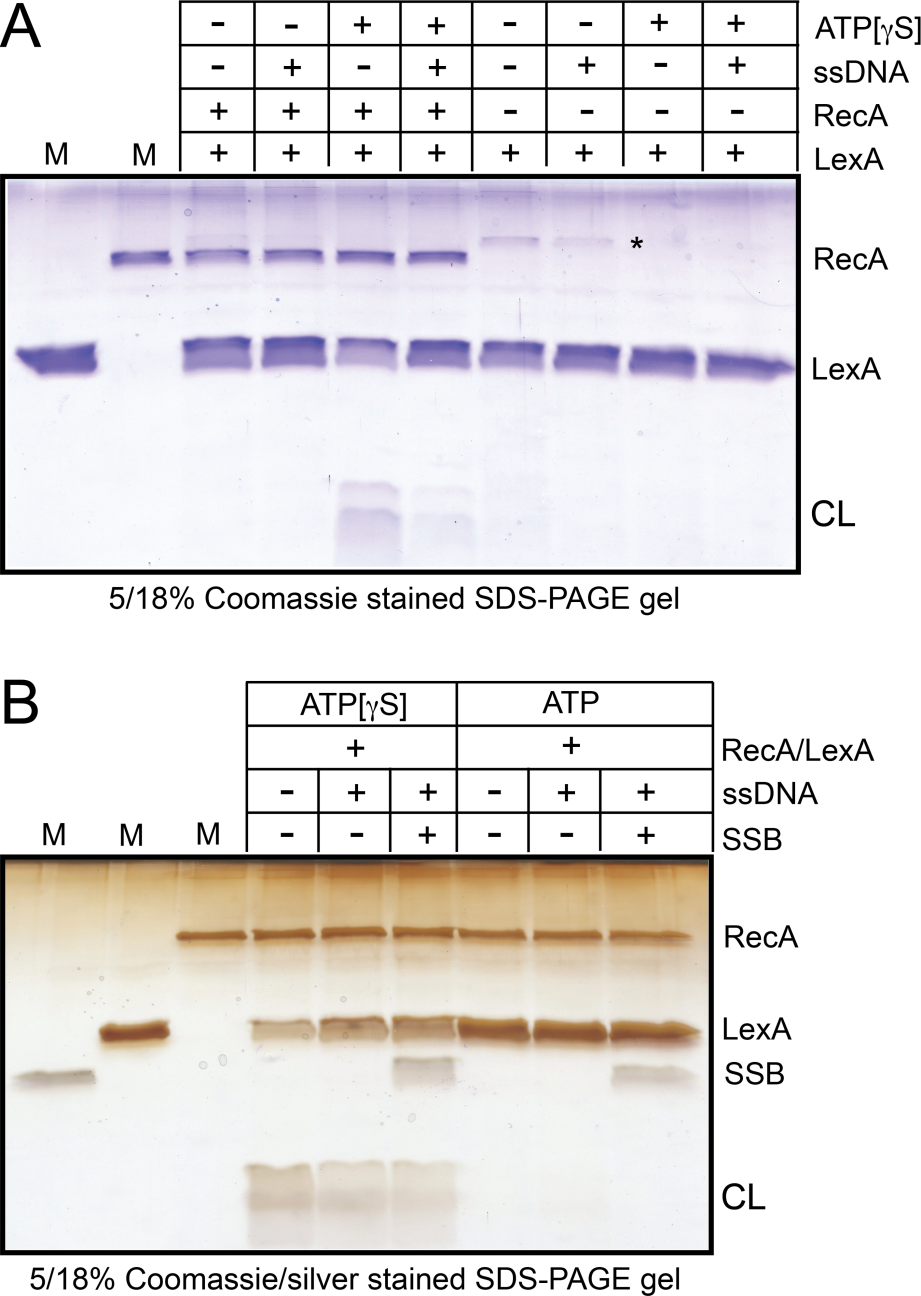
*B*. *burgdorferi* RecA possesses a weak DNA-independent co-protease activity with *E*. *coli* LexA. A) 5/18% SDS-PAGE analysis of nucleotide and DNA requirements for co-protease activity with *E*. *coli* LexA. Reactions were performed as detailed in the Materials and methods by incubation of RecA/LexA or LexA with the buffer components indicated in the gel-loading key at 30°C for 16 h. RecA was present at 1.25 μM, LexA at 1.5 μM, and where indicated, donor ssDNA was present in excess of RecA (2 μM nt). The gel was visualized by Coomassie Brilliant Blue staining. M denotes loading controls for each protein to mark their gel-migration position, CL denotes the LexA cleavage fragments. The asterisk in somes lanes of the LexA only incubation controls corresponds to the migration position of a LexA dimer. We infer that some LexA dimers formed during the overnight incubation (through cysteine oxidation) and were not completely disrupted before gel loading. B) 5/18% SDS-PAGE analysis of the effect of supplementing co-protease reactions with *B*. *burgdorferi* SSB. RecA was present at 1.25 μM, LexA at 1.5 μM, SSB at 0.19 μM and where indicated donor ssDNA was present at 0.5 μM (nt). To aid visualization of low abundance SSB the gel was silver stained subsequent to Coomassie Brilliant Blue staining. M denotes loading controls for each protein to mark their gel-migration position, CL denotes the LexA cleavage fragments.

A key biological role of the RecA presynaptic filament in many eubacteria is to signal the presence of DNA damage by acting as a coprotease for the transcriptional repressor LexA [4]. Besides being a transcriptional repressor that silences SOS response genes, LexA is a self-cleaving serine protease chaperoned to cleave itself by the activated form of RecA (the presynaptic filament), thereby, activating the SOS response genes [43,44]. *B*. *burgdorferi* is an obligate pathogen that has undergone genome reduction and, apparently, does not possess an SOS response [19,21,35]. Despite the lack of a LexA homologue and a DNA damage-stimulated SOS response, genetic complementation of *E*. *coli recA* mutants with *B*. *burgdorferi recA* has implied that *B*. *burgdorferi* RecA must possess coprotease activity with *E*. *coli* LexA and λ repressor [18]. Therefore, we purified *E*. *coli* LexA and tested *B*. *burgdorferi* RecA’s ability to support LexA self-cleavage (Fig 8). We found *B*. *burgdorferi* RecA does possess a weak coprotease activity that promotes slow LexA self-cleavage under conditions where LexA, on its own, does not self-cleave (Fig 8A). The coprotease activity is slow, producing significant LexA cleavage in overnight reactions incubated at 30°C. The coprotease activity requires ATP[γS] to be used as the nucleotide cofactor; neither AMPPNP nor ATP support coprotease activity (data not shown). An unusual property of the coprotease activity was its DNA-independence. Addition of excess amounts ssDNA actually reduced the yield of LexA cleavage products (Fig 8A). This implies that *B*. *burgdorferi* RecA can assume an activated filament form without DNA when ATP[γS] is present in the buffer. This is, perhaps, akin to the observation that *E*. *coli* RecA can be activated without DNA in high salt conditions that promote the formation of RecA aggregates [45]. The ssDNA-independence of the coprotease activity could explain why in complementation of an *E*. *coli recA* strain, induction of DNA damage was not required to induce a λ lysogen [18]. *E*. *coli* RecA’s coprotease activity is stimulated by SSB, therefore, we also tested if *B*. *burgdorferi* RecA’s coprotease activity was affected by addition of SSB (Fig 8B). Consistent with the DNA-independence of the coprotease activity addition of cognate SSB had no effect on the coprotease activity, either in conditions with a static filament (ATP[γS]) or with ATP as the nucleotide cofactor (Fig 8B). The purified RecA from another obligate pathogen that lacks a canonical LexA homologue and SOS response, *Neisseria gonnorrhoea*, has also been shown to possess coprotease activity with *E*. *coli* LexA [41,46]. *In vivo*, plausible coprotease targets could be transcriptional repressors encoded by the cp32 family of circular plasmids that have been shown to be the prophage of a transducing phage [47,48]. These lysogens can be moderately induced to make phage progeny by use of the DNA damaging agent 1-methyl-3-nitro-1-nitrosoguanidine (MNNG) implying a possible role of RecA coprotease activity in their induction [48].

## Conclusions

In this study we have biochemically characterized the RecA protein from *Borrelia burgdorferi*. We find it to have most of the properties of previously characterized RecA proteins. We find the *B*. *burgdorferi* enzyme to be a slower DNA-dependent ATPase and strand exchange protein than its *E*. *coli* counterpart. *B*. *burgdorferi* RecA showed an unexpected dependence of strand pairing and exchange reactions on the presence of SSB. Additionally, we infer an enhanced requirement, *in vivo*, for a recombinational mediator to load RecA to ssDNA that has been prebound by SSB since prebinding SSB to ssDNA had a strong inhibitory effect on *in vitro* reactions. We conclude that the unusual phenotype of *recA* mutation in *B*. *burgdorferi* is mostly due to the absence of an SOS response, not due to any intrinsic property of its RecA.

The slower DNA-dependent ATPase and strand exchange activity of *B*. *burgdorferi’*s RecA relative to *E*. *coli*’s RecA may be well tolerated since *B*. *burgdorferi*’s generation time *burgdorferi* is ~12-fold longer than that of *E*. *coli*. It has been noted by comparing the ATPase and strand exchange activities of T4 UvsX, *E*. *coli* RecA and yeast Rad51 that as the genome the recombinase processes becomes larger and more complex and as the generation time of the organism increases, the ATPase and strand exchange activities of the recombinase becomes slower [6].

The reason for the unusual dependence of *B*. *burgdorferi* RecA on SSB for DNA pairing and strand exchange, even with short substrate DNAs, is not currently understood. We note that the dependence on SSB occurs under conditions that minimize ssDNA secondary structures and after RecA binding to ssDNA, so SSB is likely not playing a role in RecA pre-synaptic filament formation by disrupting inhibitory secondary structures. Instead, a primarily post-synaptic role in sequestering the displaced strand during strand invasion and exchange is most likely. An enhanced need, relative to *E*. *coli* RecA, for sequestration of the displaced strand indicates that the reaction promoted by *B*. *burgdorferi* RecA is more prone to reversal if the displaced strand is not sequestered. Precedent for this role for a single-stranded DNA binding protein is found in studies with yeast RPA and Rad51 [36]. We note that the availability of both proteins will be important determinants of the ability to recombine homologous DNAs or to repair DNA double-strand breaks and stalled forks *in vivo*. The typical cellular concentration of SSB and RecA in *B*. *burgdorferi* is currently unknown.

We note that SSB binds ssDNA faster than RecA and that pre-binding ssDNA strongly inhibits *B*. *burgdorferi* RecA’s strand pairing and exchange activities. Therefore, *in vivo*, there should be an enhanced requirement for a recombinational mediator to facilitate SSB-RecA exchange. This role will have to be played by a non-canonical pathway in *B*. *burgdorferi* since the RecFOR complex that normally plays this role in eubacteria is absent [16,35].

Finally, it seems possible that *B*. *burgdorferi* may have an alternative recombinase that obscures some of the expected phenotypes *recA* mutation. The hypothetical alternative recombinase is envisioned as being able to play a role in replication restart, explaining why *recA* null mutants show no attenuation of growth rate and viability and to play a role in *vlsE* antigenic variation, since this process appears to produce heteroduplex DNA (branch migrated by RuvAB) but to be independent of RecA [19,20,22]. Bioinformatics has not revealed any candidate ATP-dependent RecA-like alternative recombinase but several paralogs of a putative ERF family single-strand annealing recombinase have been noted on the cp32 plasmids [49] and a purified member has been shown to possess single-strand DNA binding activity [50]. Additionally, ResT, the telomere resolvase has been shown to possess single-strand annealing activity [16,51]. Single-strand annealing proteins can promote recombination and double-strand break repair via non-conservative pathways [52].

## Supporting information

S1 Fig Alignment of *B*. *burgdorferi* and *E*. *coli* RecA proteins. RecA sequences from *E*. *coli* K12 (UniProtKB accession # P0A7G6) and *B*. *burgdorferi* B31 (GenBank accession # AAC66507) aligned. Amino acid identities are indicated with coloured boxes. The Zappo colouring scheme that groups amino acids by their physiochemico properties was followed [53]. Boxed in red are the Walker A and Walker B boxes, underlined are L1 and L2 DNA binding loops and the Walker A residue mutated in the study is shown with an asterisk. The alignment was performed using the Protein Figure program of the Sequence Manipulation Suite (http://www.bioinformatics.org/sms/; [54]).
(TIF)

S2 Fig Alignment of *B*. *burgdorferi* and *E*. *coli* SSB proteins. SSB sequences from *E*. *coli* K12 (UniProteKB accession # P0AGE0.2) and *B*. *burgdorferi* B31 (GenBank accession # AAC66492.1) aligned. Amino acid identities are indicated with coloured boxes. The Zappo colouring scheme that groups amino acids by their physiochemico properties was followed [53]. The alignment was performed using the Protein Figure program of the Sequence Manipulation Suite (http://www.bioinformatics.org/sms/; [54]).
(TIF)

S3 Fig RecA (K88R) binds ATP and has reduced DNA-dependent ATPase activity. A) 12% SDS-PAGE analysis of RecA (K88R) ATP photoaffinity binding assays.
B) Summary of ATPase assay results with the indicated concentrations of RecA -/+ 10 μg/mL ϕX174 virion (ssDNA) and ϕX174 RF1 (dsDNA). ATPase assays containing 1 μM RecA were incubated at 37°C for 120 min for ϕX174 virion (ssDNA) containing reactions or at 37°C for 120 min for DNA-free and ϕX174 RF1 (dsDNA) containing reactions. The mean and standard deviation of at least three independent experiments is shown.
C) Summary of ATPase assay results with RecA vs. RecA (K88R) -/+ 10 μg/mL ϕX174 virion DNA. ATPase assays containing 1 μM wild type RecA were incubated at 37°C for 30 min for ϕX174 virion (ssDNA) containing reactions or at 37°C for 120 min for DNA-free containing reactions. ATPase assays containing 1 μM RecA (K88R) were incubated at 37°C for 120 min for both DNA-free and ssDNA containing reactions. The mean and standard deviation of at least three independent experiments is shown.
(TIF)

S4 Fig ATP regeneration does not alter the dynamics of strand invasion. 0.8% agarose 1X TAE gel analysis of D-loop formation conducted -/+ the presence of an ATP regeneration system. RecA was present at 2 μM, donor ssDNA at 1.9 μM (nt) and pUC19 at 77 μM (nt). Where indicated, SSB was present at 300 nM and was added after pre-incubation of RecA with the donor ssDNA. The ATP regeneration system consisted of 3.3 mM phosphoenolpyruvate (PEP) and 10 units/mL pyruvate kinase.
(TIF)

S5 Fig SSB loads to single-stranded donor DNA before RecA. A) Schematic representation of the strand invasion assay indicating the effect of the order of SSB/RecA addition and ATP hydrolysis on D-loop recovery.
B) 0.8% agarose 1X TAE gel analysis of strand invasion timecourses performed with 0.3 μM SSB and 2 μM RecA in buffer containing ATP (left panel) or AMPPNP (right panel).
D-loop formation was assayed in a buffer containing 25 mM HEPES (pH 7.6), 2 mM MgCl_2_, 1 mM DTT, 100 μg/ml BSA, 50 mM NaCl and 2 mM ATP or AMPPNP in a 120 μL reaction volume. The assay was conducted as a staged reaction with pre-incubation of SSB, RecA or both SSB and RecA simultaneously with the donor ssDNA (30°C, 5 min) followed by addition of SSB or RecA (where required) and supercoiled pUC19 target plasmid and continued incubation at 37°C for the indicated times.
(TIF)

S6 Fig RecA (K88R) promotes strand exchange with oligonucleotide substrates when supplemented with SSB. A) Schematic representation of the oligonucleotide substrates used. The ssDNA donor oligonucleotide is 70 nt in length while the 5’-endlabeled duplex target DNA is 35 bp. * denote the ^32^P-endlabels, red/shaded lines represent endlabeled strands.B) 8% PAGE 1X TAE/0.1% SDS gel analysis of strand exchange reactions with RecA (K88R) and SSB. RecA was present at 2 μM, donor ssDNA at 1.4 μM (nt) and target duplex at 1.4 μM (nt). When added, SSB was added after pre-incubation of RecA with the single stranded donor and was present at 300 nM. The proteins and nucleotide co-factor present are indicated in the loading key above the gels.
(TIF)

S7 Fig *E*. *coli* RecA promoted strand exchange with oligonucleotide substrates. 8% PAGE 1X TAE/0.1% SDS gel analysis of strand exchange reactions with *E*. *coli* RecA and SSB. RecA was present at 2 μM, donor ssDNA at 1.4 μM (nt) and target duplex at 1.4 μM (nt). When added, SSB was added after pre-incubation of RecA with the single stranded donor and was present at 300 nM. The proteins and nucleotide co-factor present are indicated in the loading key above the gels.
(TIF)

S8 Fig *E*. *coli* RecA promoted strand exchange with long substrates. 0.8% agarose 1X TAE gel analysis of strand exchange reactions performed with *E*. *coli* RecA and SSB and a buffer containing 2 mM ATP. RecA was present at 2 μM, ϕX174 virion (css) at 5.1 μM (nt) and XhoI-linearized ϕX174 duplex DNA (lds) at 5.1 μM (nt). SSB was added after pre-incubation of RecA with the single stranded donor and was present at 0.9 μM. The concentration of added MgCl_2_ is noted in the loading key above the gel. The migration position of the substrate DNA is noted to the left of the gel and of the products to the right. Under our gel conditions css and lss have identical gel mobilities.
(TIF)

S1 Protocol Purification of *E*. *coli* LexA. (DOCX)

S1 Sequence Codon-optimized *B*. *burgdorferi* RecA synthetic gene sequence. (DOCX)

S1 Table Oligonucleotides used in this study. (DOCX)
