# Supplementary figures and images for: Biochemical characterization of Borrelia burgdorferi’s RecA protein
Source: PLoS One. 2017 Oct 31;12(10):e0187382. doi: 10.1371/journal.pone.0187382 (PMC5663514; doi:10.1371/journal.pone.0187382)

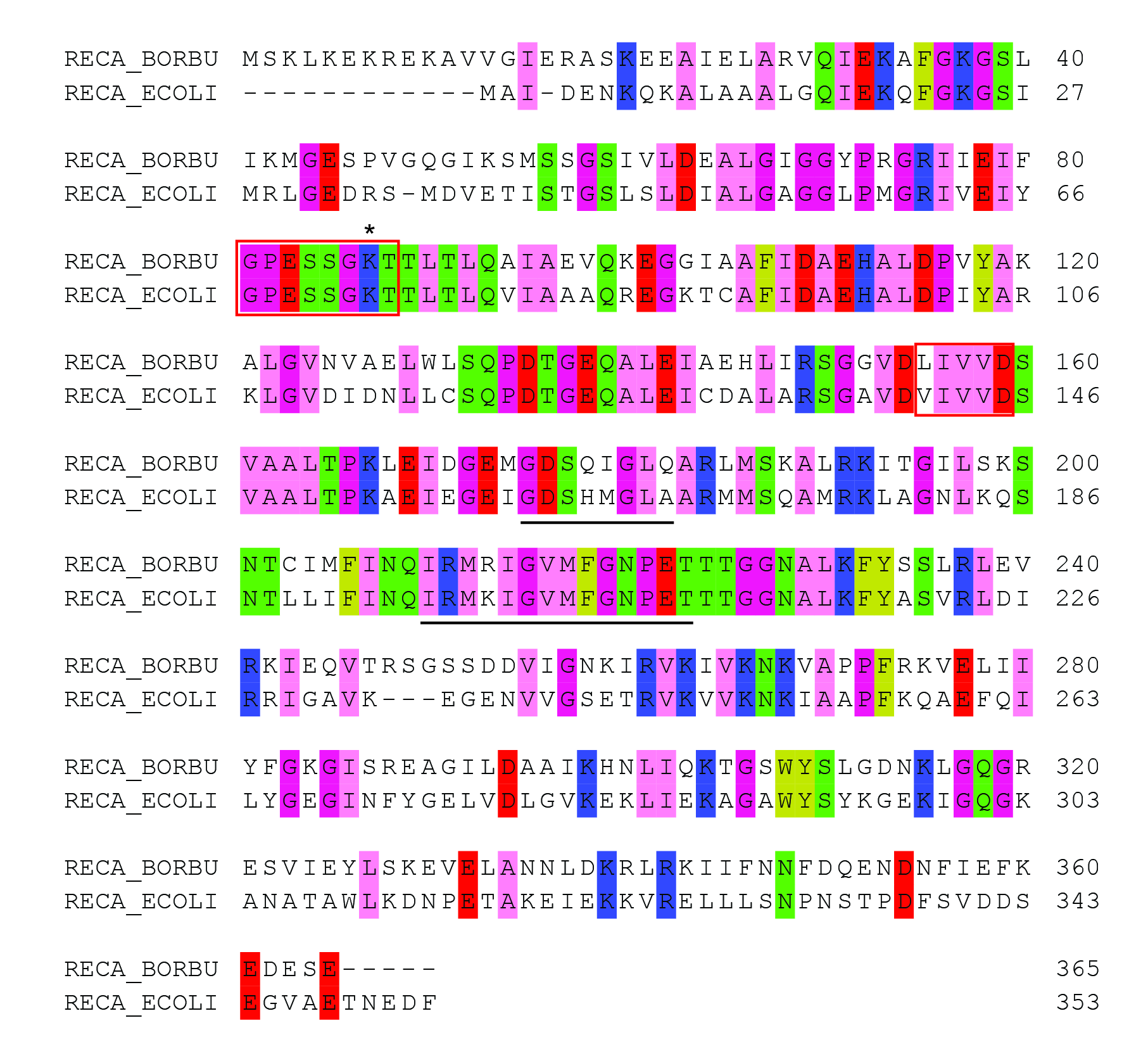

Supplement: S1 Fig — RecA sequences from E. coli K12 (UniProtKB accession # P0A7G6) and B. burgdorferi B31 (GenBank accession # AAC66507) aligned. Amino acid identities are indicated with coloured boxes. The Zappo colouring scheme that groups amino acids by their physiochemico properties was followed [53]. Boxed in red are the Walker A and Walker B boxes, underlined are L1 and L2 DNA binding loops and the Walker A residue mutated in the study is shown with an asterisk. The alignment was performed using the Protein Figure program of the Sequence Manipulation Suite (http://www.bioinformatics.org/sms/; [54]). (TIF) [file pone.0187382.s001.tif]

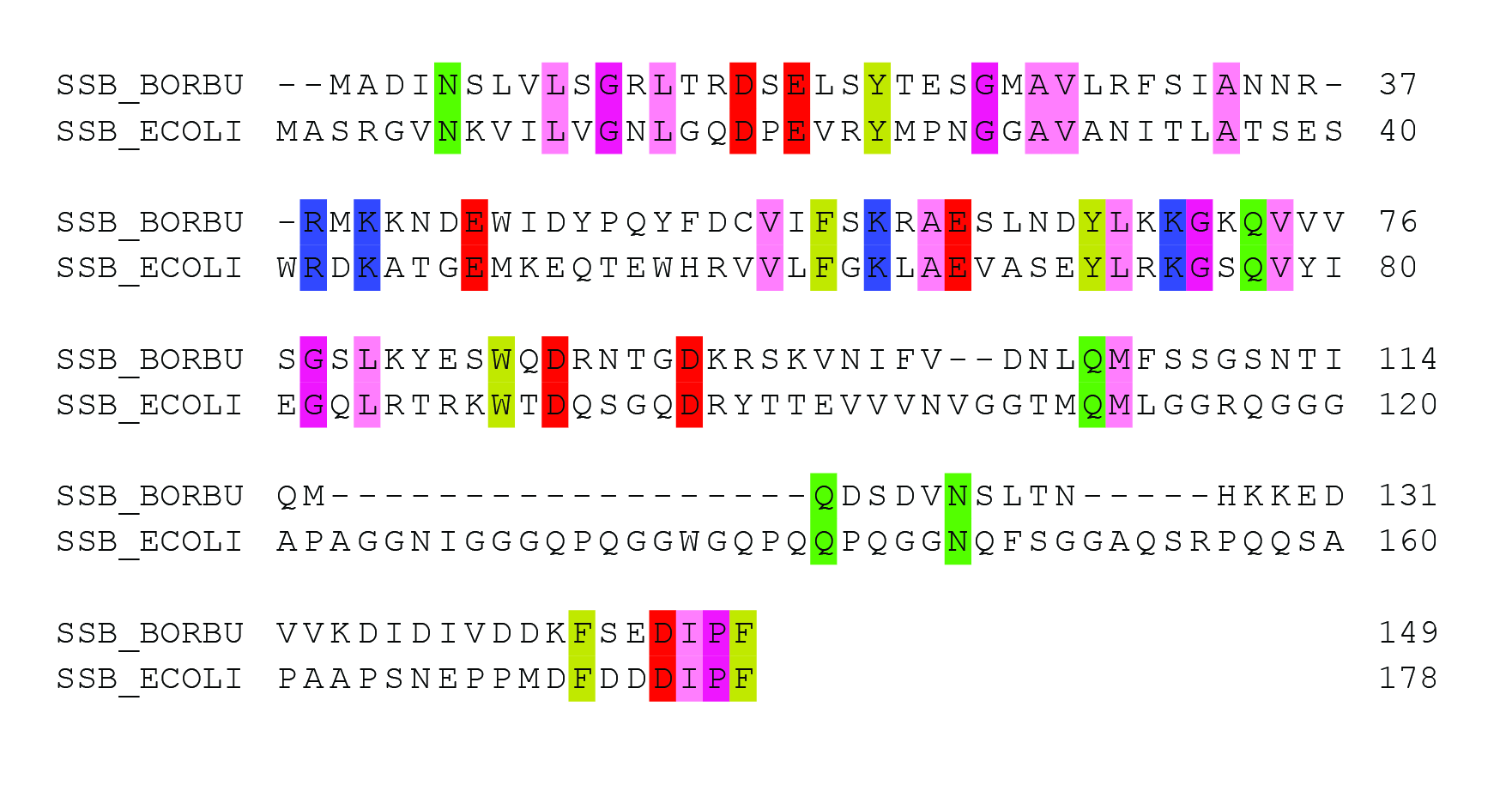

Supplement: S2 Fig — SSB sequences from E. coli K12 (UniProteKB accession # P0AGE0.2) and B. burgdorferi B31 (GenBank accession # AAC66492.1) aligned. Amino acid identities are indicated with coloured boxes. The Zappo colouring scheme that groups amino acids by their physiochemico properties was followed [53]. The alignment was performed using the Protein Figure program of the Sequence Manipulation Suite (http://www.bioinformatics.org/sms/; [54]). (TIF) [file pone.0187382.s002.tif]

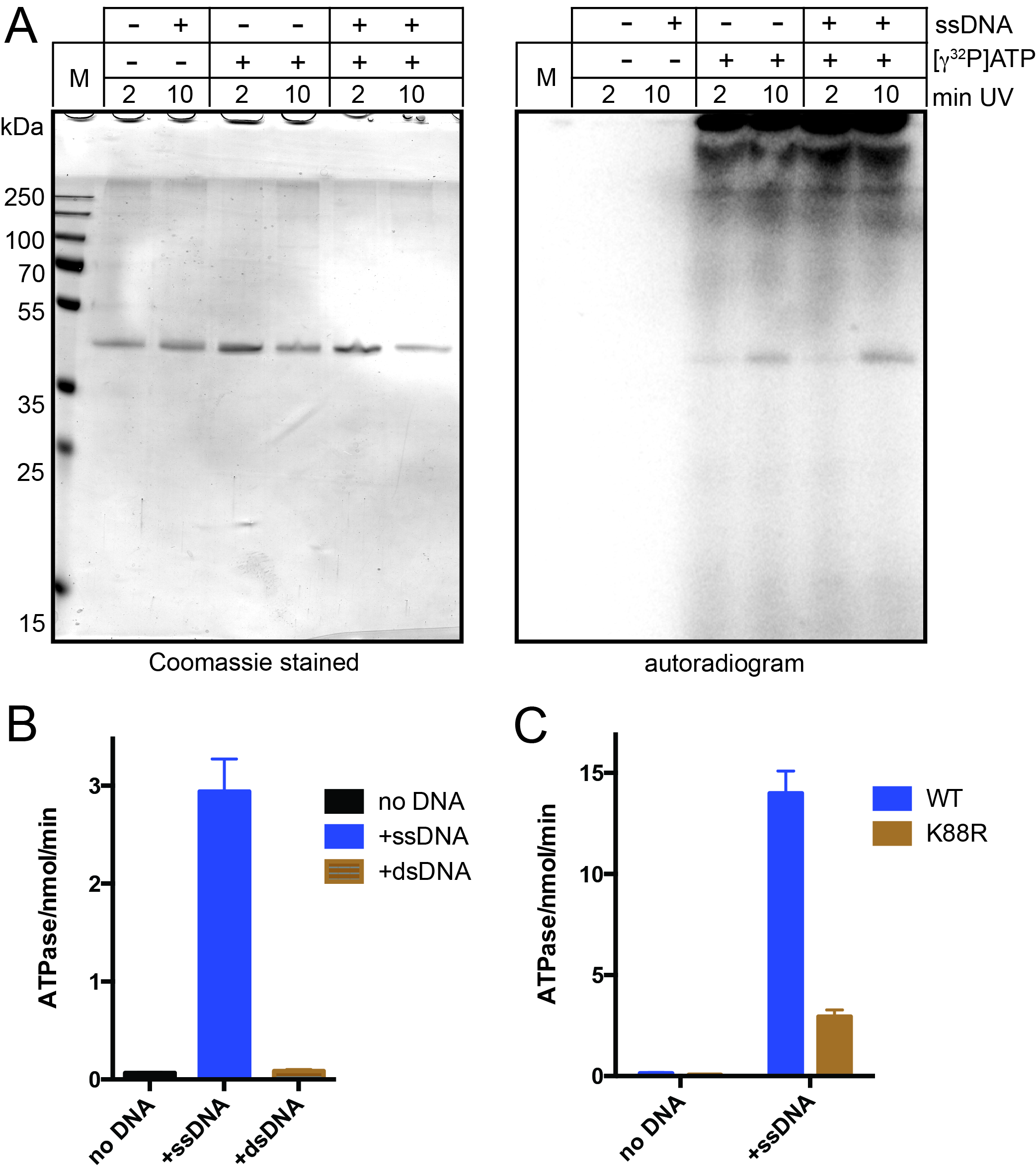

Supplement: S3 Fig — A) 12% SDS-PAGE analysis of RecA (K88R) ATP photoaffinity binding assays. B) Summary of ATPase assay results with the indicated concentrations of RecA -/+ 10 μg/mL ϕX174 virion (ssDNA) and ϕX174 RF1 (dsDNA). ATPase assays containing 1 μM RecA were incubated at 37°C for 120 min for ϕX174 virion (ssDNA) containing reactions or at 37°C for 120 min for DNA-free and ϕX174 RF1 (dsDNA) containing reactions. The mean and standard deviation of at least three independent experiments is shown. C) Summary of ATPase assay results with RecA vs. RecA (K88R) -/+ 10 μg/mL ϕX174 virion DNA. ATPase assays containing 1 μM wild type RecA were incubated at 37°C for 30 min for ϕX174 virion (ssDNA) containing reactions or at 37°C for 120 min for DNA-free containing reactions. ATPase assays containing 1 μM RecA (K88R) were incubated at 37°C for 120 min for both DNA-free and ssDNA containing reactions. The mean and standard deviation of at least three independent experiments is shown. (TIF) [file pone.0187382.s003.tif]

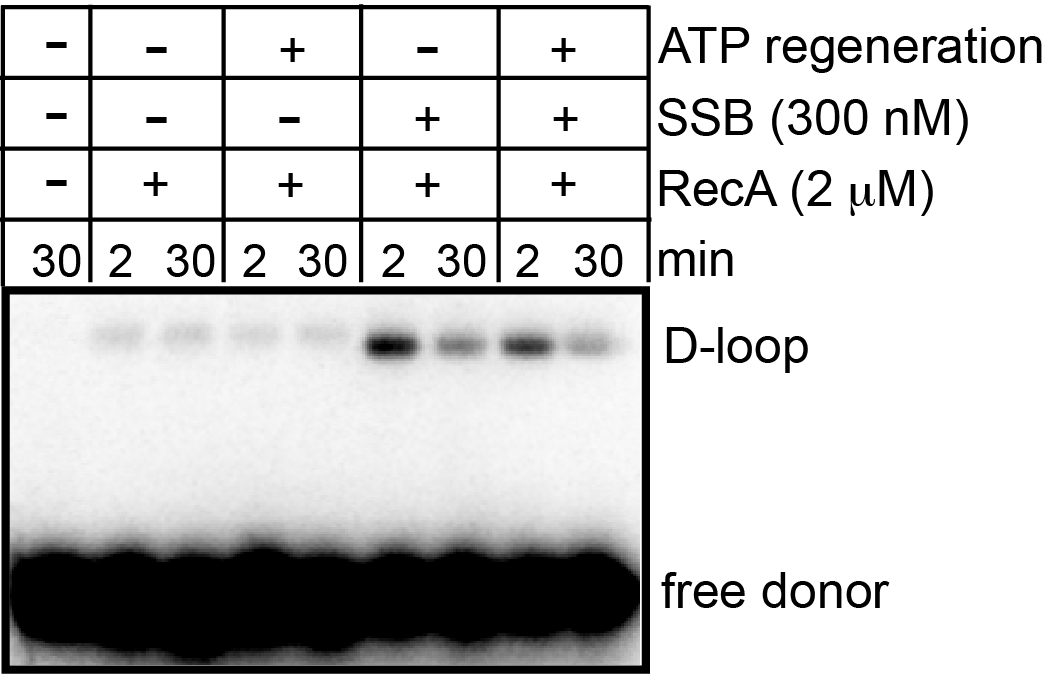

Supplement: S4 Fig — 0.8% agarose 1X TAE gel analysis of D-loop formation conducted -/+ the presence of an ATP regeneration system. RecA was present at 2 μM, donor ssDNA at 1.9 μM (nt) and pUC19 at 77 μM (nt). Where indicated, SSB was present at 300 nM and was added after pre-incubation of RecA with the donor ssDNA. The ATP regeneration system consisted of 3.3 mM phosphoenolpyruvate (PEP) and 10 units/mL pyruvate kinase. (TIF) [file pone.0187382.s004.tif]

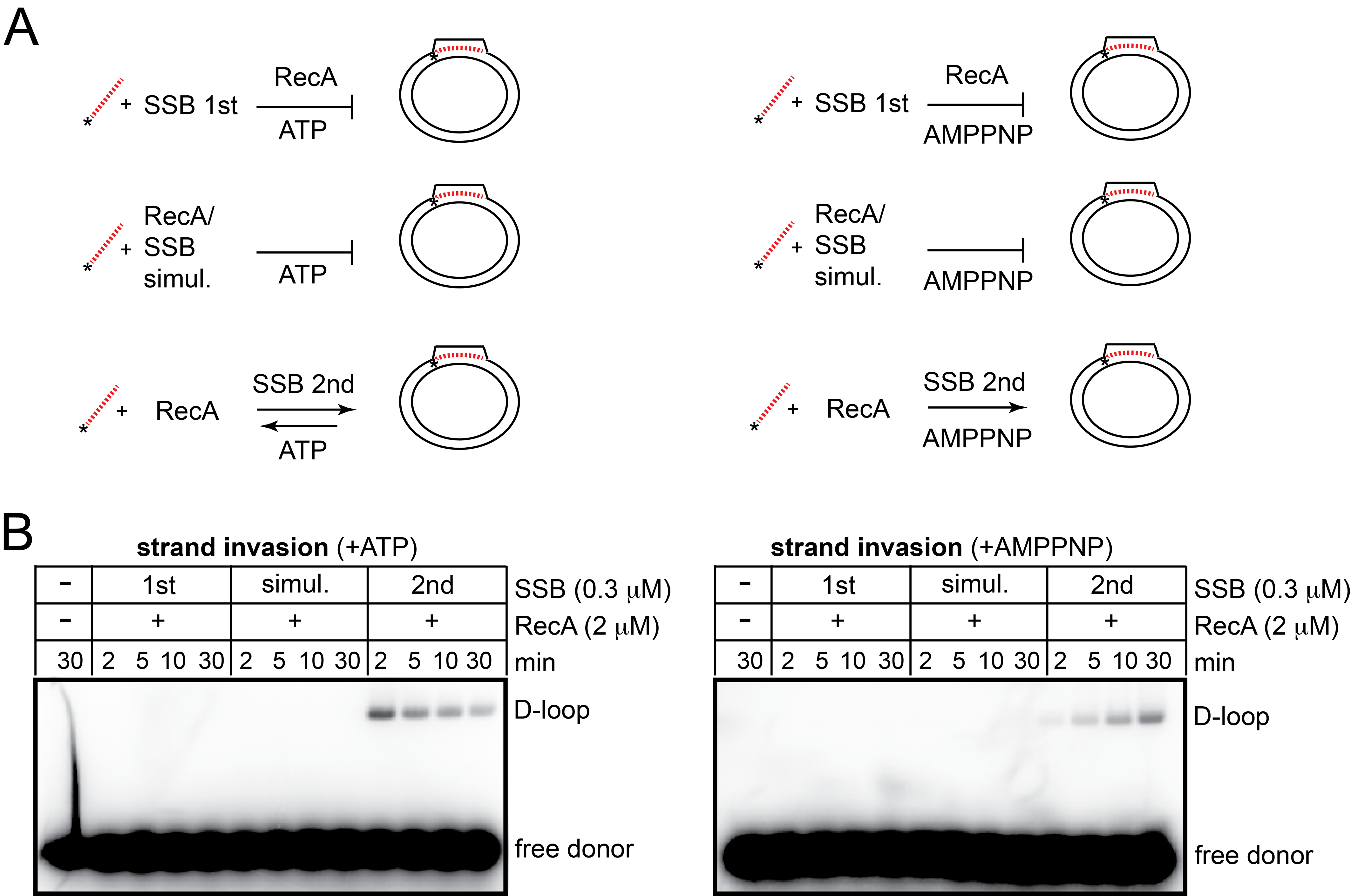

Supplement: S5 Fig — A) Schematic representation of the strand invasion assay indicating the effect of the order of SSB/RecA addition and ATP hydrolysis on D-loop recovery. B) 0.8% agarose 1X TAE gel analysis of strand invasion timecourses performed with 0.3 μM SSB and 2 μM RecA in buffer containing ATP (left panel) or AMPPNP (right panel). D-loop formation was assayed in a buffer containing 25 mM HEPES (pH 7.6), 2 mM MgCl2, 1 mM DTT, 100 μg/ml BSA, 50 mM NaCl and 2 mM ATP or AMPPNP in a 120 μL reaction volume. The assay was conducted as a staged reaction with pre-incubation of SSB, RecA or both SSB and RecA simultaneously with the donor ssDNA (30°C, 5 min) followed by addition of SSB or RecA (where required) and supercoiled pUC19 target plasmid and continued incubation at 37°C for the indicated times. (TIF) [file pone.0187382.s005.tif]

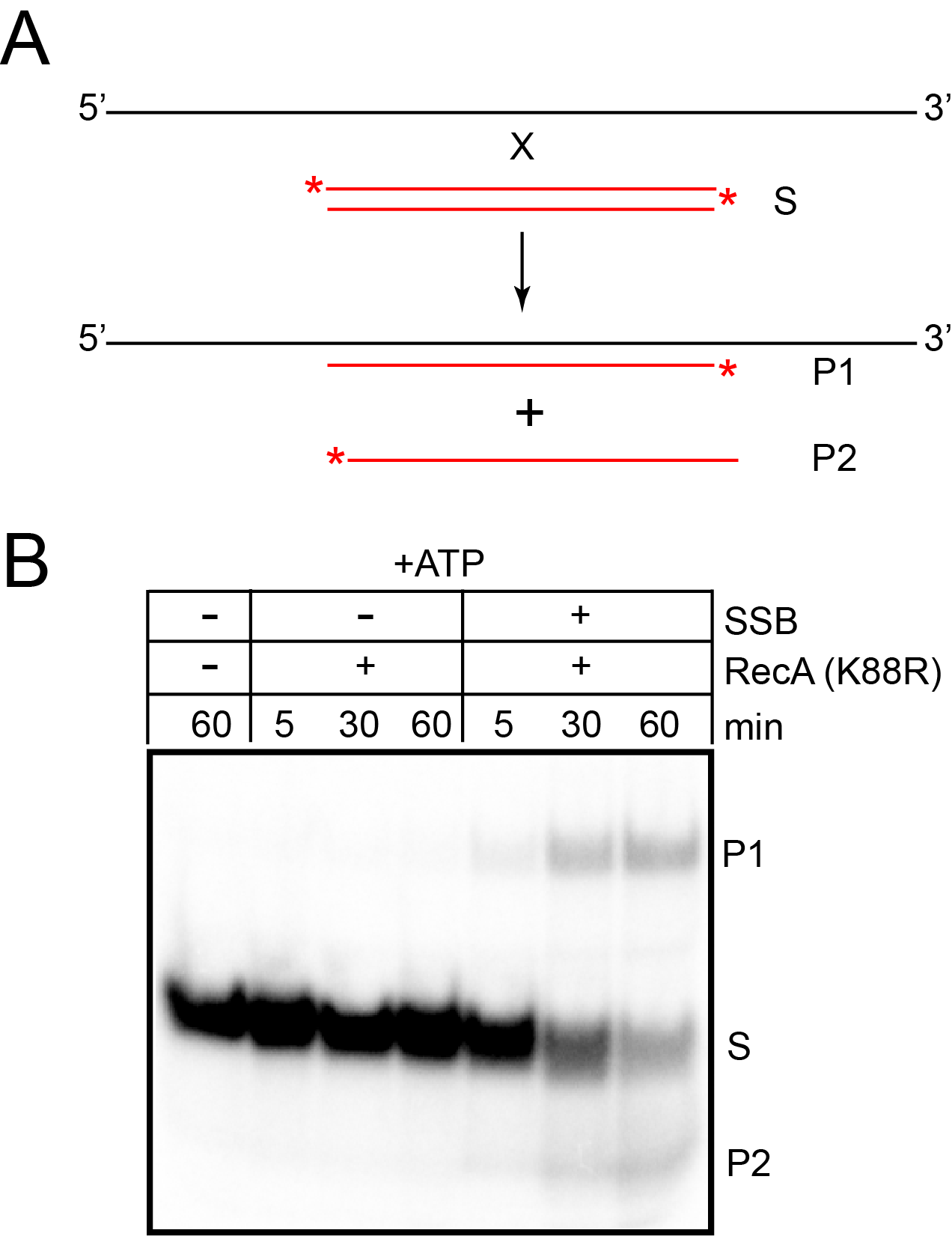

Supplement: S6 Fig — A) Schematic representation of the oligonucleotide substrates used. The ssDNA donor oligonucleotide is 70 nt in length while the 5’-endlabeled duplex target DNA is 35 bp. * denote the 32P-endlabels, red/shaded lines represent endlabeled strands.B) 8% PAGE 1X TAE/0.1% SDS gel analysis of strand exchange reactions with RecA (K88R) and SSB. RecA was present at 2 μM, donor ssDNA at 1.4 μM (nt) and target duplex at 1.4 μM (nt). When added, SSB was added after pre-incubation of RecA with the single stranded donor and was present at 300 nM. The proteins and nucleotide co-factor present are indicated in the loading key above the gels. (TIF) [file pone.0187382.s006.tif]

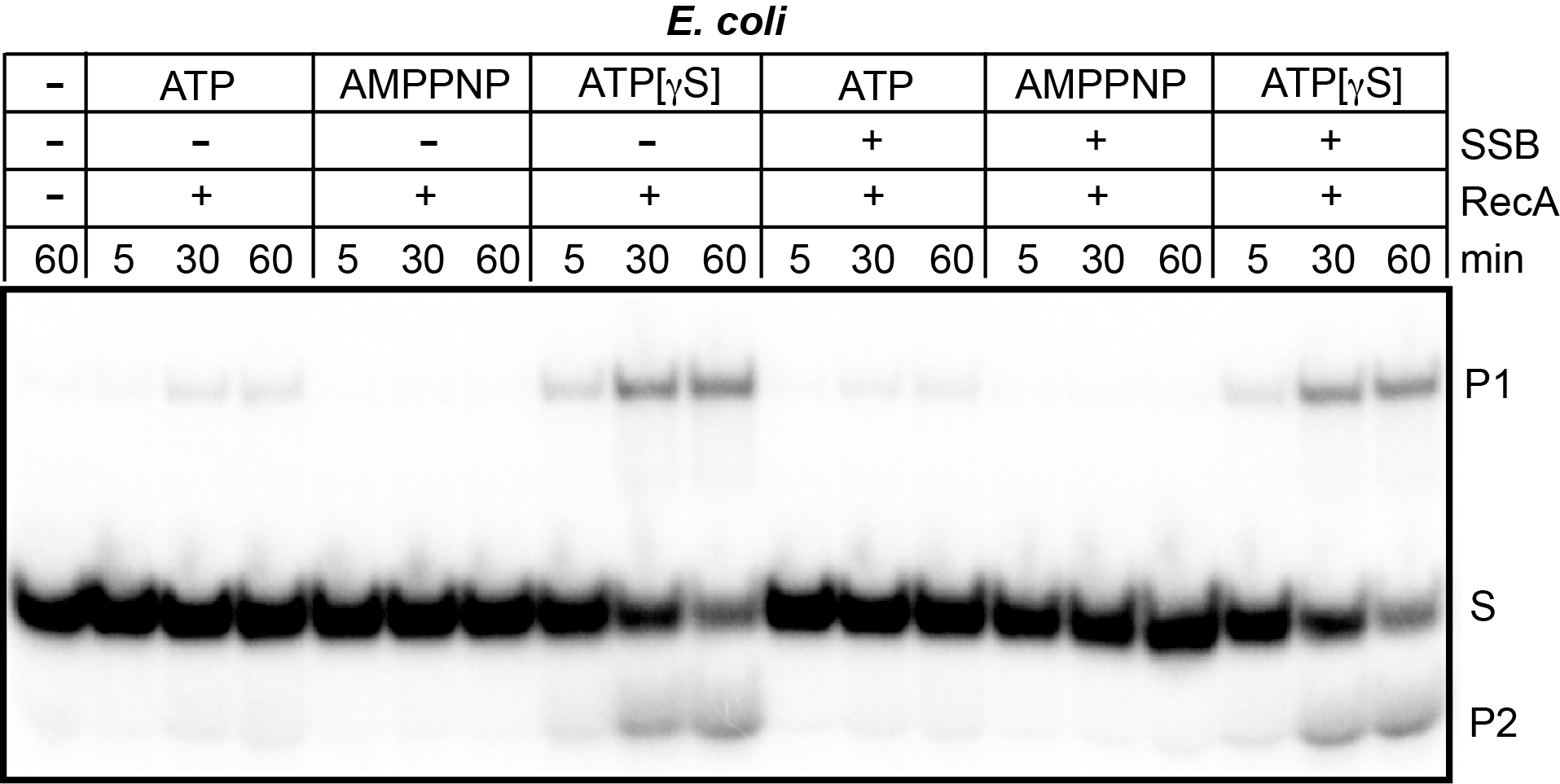

Supplement: S7 Fig — 8% PAGE 1X TAE/0.1% SDS gel analysis of strand exchange reactions with E. coli RecA and SSB. RecA was present at 2 μM, donor ssDNA at 1.4 μM (nt) and target duplex at 1.4 μM (nt). When added, SSB was added after pre-incubation of RecA with the single stranded donor and was present at 300 nM. The proteins and nucleotide co-factor present are indicated in the loading key above the gels. (TIF) [file pone.0187382.s007.tif]

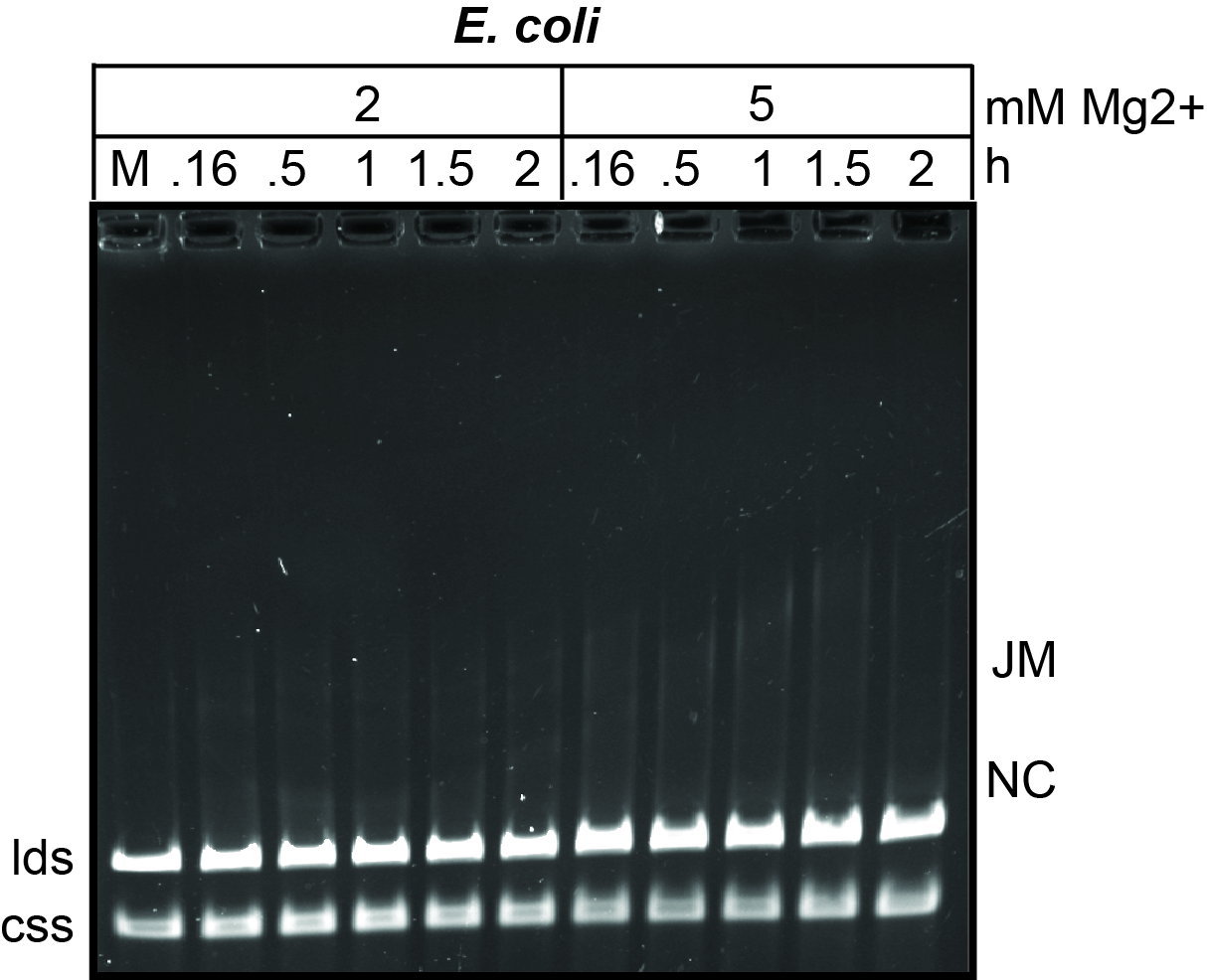

Supplement: S8 Fig — 0.8% agarose 1X TAE gel analysis of strand exchange reactions performed with E. coli RecA and SSB and a buffer containing 2 mM ATP. RecA was present at 2 μM, ϕX174 virion (css) at 5.1 μM (nt) and XhoI-linearized ϕX174 duplex DNA (lds) at 5.1 μM (nt). SSB was added after pre-incubation of RecA with the single stranded donor and was present at 0.9 μM. The concentration of added MgCl2 is noted in the loading key above the gel. The migration position of the substrate DNA is noted to the left of the gel and of the products to the right. Under our gel conditions css and lss have identical gel mobilities. (TIF) [file pone.0187382.s008.tif]
