# Supplementary material for: Biochemical characterization of Borrelia burgdorferi’s RecA protein
Source: PLoS One. 2017 Oct 31;12(10):e0187382. doi: 10.1371/journal.pone.0187382 (PMC5663514; doi:10.1371/journal.pone.0187382)
Supplement: S1 Protocol — (DOCX) [file pone.0187382.s009.docx]

**S1 protocol.** Purification of *E. coli* LexA.

*E. coli* LexA was purified as an N-terminally His-tagged fusion protein, essentially as reported in [1] with the modifications noted below. Instead of removing nucleic acids by PEI precipitation followed by ammonium sulphate precipitation and dialysis, the lysate was applied directly to an Ni-NTA column and a heparin sulphate column was used to complete the purification. Ni-NTA peak fractions were pooled and the NaCl concentration was adjusted from 0.5M NaCl to 0.25M NaCl by dilution with buffer HG (25 mM HEPES (7.6), 0.1 mM EDTA, 10% glycerol). A 4 mL heparin sepharose column was equilibrated with 10 column volumes of HG 0.25M NaCl and the pooled, adjusted Ni-NTA peak fractions were applied. The heparin column was washed with 5 column volumes of HG 0.25M NaCl, 2 column volumes of HG 0.4M NaCl and was eluted with 3 column volumes of HG 1.5M NaCl. Peak heparin sepharose fractions were assessed by running aliquots of each fraction on 5/18% SDS-PAGE gels.

1. Luo Y, Pfuetzner RA, Mosimann S, Paetzel M, Frey EA, et al. (2001) Crystal structure of LexA: a conformational switch for regulation of self-cleavage. Cell 106: 585-594.
