# Supplementary material for: Biochemical characterization of Borrelia burgdorferi’s RecA protein
Source: PLoS One. 2017 Oct 31;12(10):e0187382. doi: 10.1371/journal.pone.0187382 (PMC5663514; doi:10.1371/journal.pone.0187382)
Supplement: S1 Sequence — (DOCX) [file pone.0187382.s010.docx]

**S1 sequence.** Codon-optimized *B. burgdorferi* RecA synthetic gene sequence.

ATG AGC AAA CTT AAA GAG AAA CGT GAA AAA GCG GTG GTG GGG ATC GAG CGG GCG TCT AAA GAG GAA GCG ATC GAA CTG GCG CGC GTT CAG ATC GAA AAG GCG TTT GGT AAA GGC TCA CTG ATT AAA ATG GGT GAA TCT CCA GTC GGA CAG GGC ATT AAA TCG ATG AGC TCG GGC AGC ATT GTG CTG GAC GAA GCA CTG GGT ATT GGC GGC TAC CCC CGC GGC CGT ATT ATC GAA ATC TTC GGC CCA GAA AGC TCC GGT AAG ACT ACA TTA ACG CTT CAG GCG ATT GCA GAG GTG CAA AAG GAA GGT GGT ATT GCT GCG TTT ATT GAC GCA GAA CAT GCA CTT GAC CCT GTT TAC GCA AAA GCA CTG GGA GTT AAC GTT GCG GAG TTA TGG CTG TCC CAG CCT GAC ACT GGG GAG CAA GCC CTT GAA ATT GCA GAA CAT TTA ATC CGC AGC GGC GGC GTC GAT TTG ATT GTT GTT GAC TCC GTT GCA GCA CTC ACG CCA AAA CTG GAG ATT GAT GGA GAA ATG GGG GAT AGC CAG ATT GGA CTG CAG GCC CGT TTG ATG TCA AAA GCC CTG CGT AAG ATT ACA GGA ATT CTT AGT AAA AGT AAC ACT TGC ATT ATG TTT ATT AAC CAG ATT CGC ATG CGC ATC GGT GTC ATG TTT GGC AAC CCC GAA ACC ACC ACC GGC GGT AAT GCT CTG AAG TTC TAT TCG AGC CTG CGC TTA GAA GTC CGT AAA ATT GAA CAA GTC ACG CGT TCA GGC TCT AGC GAT GAC GTT ATT GGG AAC AAA ATC CGT GTG AAA ATT GTA AAA AAT AAA GTT GCC CCG CCC TTC CGG AAA GTG GAA CTG ATC ATT TAT TTC GGC AAA GGC ATT AGC CGC GAG GCA GGC ATT CTG GAT GCC GCG ATC AAG CAT AAT CTG ATT CAG AAA ACC GGT TCG TGG TAT AGC CTG GGT GAC AAT AAA CTC GGC CAA GGT CGT GAG TCT GTG ATC GAG TAT CTG AGC AAA GAG GTT GAA CTT GCC AAC AAC CTG GAT AAG CGC CTG CGC AAA ATC ATC TTT AAC AAC TTT GAT CAG GAA AAC GAT AAC TTT ATT GAA TTT AAA GAA GAT GAA AGC GAA
