# Supplementary material for: Biochemical characterization of Borrelia burgdorferi’s RecA protein
Source: PLoS One. 2017 Oct 31;12(10):e0187382. doi: 10.1371/journal.pone.0187382 (PMC5663514; doi:10.1371/journal.pone.0187382)
Supplement: S1 Table — (DOCX) [file pone.0187382.s011.docx]

**S1 Table.** Oligonucleotides used in this study.

| **Oligo name** | **Oligo sequence** | **Use** |
| --- | --- | --- |
| OGCB396 | 5^′^-TGCAGGCATGCAAGCTTGGCGTAATCATGGTCATAGCTGTT TCCTGTGTGAAATTGTTATCCGCTCACAATTCCACACAACATACGAGCCGGAAG-3′ | 95 nt ssDNA used in Figures 1, 3, 4 and S3. |
| OGCB742 | 5’-CTTCCGGCTCGTATGTTGTGTGGAATTGTGAGCGGATAAC AATTTCACACAGGAAACAGCTATGACCATGATTACGCCAAGCTTGCATGCCTGCA-3’ | 95 nt complement of OGC396 |
| OGCB606 | 5’-CACCATGG**CATATG**AGCAAACTTAAAGAGAAACGTGAAAA AGCGGT-3’ | N-terminal primer to clone RecA syngene into pET15b. **NdeI site bolded.** |
| OGCB607 | 5’-ATACC**GGATCC**TTATTCGCTTTCATCTTCTTTAAATTCAA TAAAGTTATCGTTTTCCT-3’ | C-terminal primer to clone RecA syngene into pET15b, stop codon added (red). **BamHI site bolded.** |
| OGCB714 | 5’-CCAGAAAGCTCCGGTAGGACTACATTAACGCTT-3’ | Mutagenic oligo used to make the K88R mutant; use with 715. |
| OGCB715 | 5’-AAGCGTTAATGTAGTCCTACCGGAGCTTTCTGG-3’ | Mutagenic oligo used to make the K88R mutant; use with 714. |
| OGCB664 | 5’-GGAAGCGATAAAACTCTGCAGGTTGGATACGCCAA-3’ | 35 nt oligo used to make 35 bp duplex; anneal with 665; used in Figures 5 and S4. |
| OGCB665 | 5’-TTGGCGTATCCAACCTGCAGAGTTTTATCGCTTCC-3’ | 35 nt oligo used to make 35 bp duplex; anneal with 664; used in Figures 5 and S4. |
| OGCB748 | 5’-TGCGCCTCGTTCCGGCTA**GGAAGCGATAAAACTCTGCA GGTTGGATACGCCAA**CGATGATACAAATCTCC-3’ | 70 nt ssDNA for strand exchange with 664/665 dsDNA (**664 identity bolded**). Used in Figures 5 and S4. |
